# MBMethPred: a computational framework for the accurate classification of childhood medulloblastoma subgroups using data integration and AI-based approaches

**DOI:** 10.3389/fgene.2023.1233657

**Published:** 2023-09-07

**Authors:** Edris Sharif Rahmani, Ankita Lawarde, Prakash Lingasamy, Sergio Vela Moreno, Andres Salumets, Vijayachitra Modhukur

**Affiliations:** ^1^ Competence Centre on Health Technologies, Tartu, Estonia; ^2^ Department of Obstetrics and Gynecology, Institute of Clinical Medicine, University of Tartu, Tartu, Estonia; ^3^ Division of Obstetrics and Gynecology, Department of Clinical Science, Intervention and Technology, Karolinska Institute and Karolinska University Hospital, Stockholm, Sweden

**Keywords:** childhood medulloblastoma, subgroup classification, DNA methylation, machine learning, gene expression, deep learning, Wnt, sonic hedgehog

## Abstract

Childhood medulloblastoma is a malignant form of brain tumor that is widely classified into four subgroups based on molecular and genetic characteristics. Accurate classification of these subgroups is crucial for appropriate treatment, monitoring plans, and targeted therapies. However, misclassification between groups 3 and 4 is common. To address this issue, an AI-based R package called MBMethPred was developed based on DNA methylation and gene expression profiles of 763 medulloblastoma samples to classify subgroups using machine learning and neural network models. The developed prediction models achieved a classification accuracy of over 96% for subgroup classification by using 399 CpGs as prediction biomarkers. We also assessed the prognostic relevance of prediction biomarkers using survival analysis. Furthermore, we identified subgroup-specific drivers of medulloblastoma using functional enrichment analysis, Shapley values, and gene network analysis. In particular, the genes involved in the nervous system development process have the potential to separate medulloblastoma subgroups with 99% accuracy. Notably, our analysis identified 16 genes that were specifically significant for subgroup classification, including *EP300*, *CXCR4, WNT4*, *ZIC4, MEIS1, SLC8A1, NFASC, ASCL2, KIF5C, SYNGAP1, SEMA4F, ROR1, DPYSL4, ARTN, RTN4RL1,* and *TLX2*. Our findings contribute to enhanced survival outcomes for patients with medulloblastoma. Continued research and validation efforts are needed to further refine and expand the utility of our approach in other cancer types, advancing personalized medicine in pediatric oncology.

## 1 Introduction

Medulloblastoma (MB) is the most prevalent malignant form of brain tumor among children, accounting for approximately 20% of all central nervous system (CNS) malignancies. The pathological features of MB are heterogeneous, and its emergence in the cerebellum is attributed to genetic and epigenetic alterations that disrupt critical pathways in cerebellar development (Northcott and Dubuc, 2012). According to the World Health Organization (WHO) classification of CNS tumors, the following four major subgroups have been identified based on molecular and genetic characteristics: wingless (WNT)-activated, sonic hedgehog (SHH)-activated, and numerically designated non-WNT/non-SHH, representing Groups 3 and 4 (Louis et al., 2016; Northcott et al., 2019; Louis et al., 2021). Accurate classification of childhood MB and its subclasses is critical for selecting appropriate treatment, monitoring plans, preventing tumor progression, and reducing mortality rates. In addition, the accurate classification of MB subgroups plays a vital role in developing targeted therapies for each specific subclass (Ramaswamy et al., 2016; Yan et al., 2020).

Advancements in multi-omics, including genomics, transcriptomics, epigenomics, and proteomics, have significantly contributed to the reporting of the biological and clinical relevance of subgroups in MB (Northcott and Dubuc, 2012; Northcott et al., 2017; Capper et al., 2018; Sharma et al., 2019)**.** Transcriptomic analysis can identify medulloblastoma subgroups, but it has limitations in capturing the microenvironment and impact of modifications on gene expression, as well as dealing with technical variations, noisy data, and incomplete transcriptome coverage. DNA methylation profiling is more reliable in accurately classifying medulloblastoma subgroups (Korshunov et al., 2017; Gomez et al., 2018). Moreover, later studies use integrative clustering methods, such as similarity network fusion, to analyze multiple data types in conjunction for improved results. However, these methods may not account for intratumor heterogeneity, which can lead to misclassification of subgroups (Northcott and Shih, 2012; Cavalli et al., 2017; Northcott et al., 2017; Alharbi et al., 2020).

Recently, various other methods have been explored for the accurate classification of medulloblastoma subgroups, including an AI-based pipeline that uses histopathological and textural images (Attallah and Zaghlool, 2022), radiomics-based machine learning models (Karabacak et al., 2022), and one-class logistic regression machine learning that integrates gene expression and DNA methylation data (Lian et al., 2019). While featuring certain limitations, such as smaller sample sizes, limited diverse datasets, and the need for high-quality images, these methods hold great potential for improving the diagnosis and treatment of medulloblastoma. The current gold standard for accurate MB subgroup classification is genome-wide transcriptional and methylation arrays, with high accuracy for WNT and SHH subgroups (Ramaswamy et al., 2016). On the other hand, classification based on immunohistochemistry (IHC) and MRI has also been utilized for subgrouping. However, the challenges associated with standardization and lack of specificity in clinical settings have limited its effectiveness (Ramaswamy et al., 2016; Yan et al., 2020). The classification of Group 3 and Group 4 tumors is particularly challenging due to their overlapping molecular features, low incidence of recurring mutations, and recurrent chromosomal alterations (Cavalli et al., 2017). To overcome this issue, integration of multi-omics data (including DNA methylation, gene expression, and clinical features) and application of machine learning algorithms for the development of accurate classification models are required (Hovestadt et al., 2020). Therefore, our study aims to develop an artificial intelligence (AI)-based framework to classify MB subgroups using publicly available DNA methylation data. Furthermore, our framework integrates DNA methylation and gene expression data. The relevance of our prediction biomarkers was further examined using Gene Ontology analysis, survival analysis, Shapley values, and network analysis.

## 2 Materials and methods

### 2.1 Data collection

We collected DNA methylation profiles of pediatric medulloblastoma patients from multiple Gene Expression Omnibus (GEO) datasets, including GSE85212 (N = 763), GSE130051 (N = 1390), GSE90496 (N = 390), GSE54880 (N = 276), GSE109379 (N = 128), and GSE75153 (N = 91) (Table 1). All the above-mentioned methylation data were profiled using the Illumina Infinium HumanMethylation450 platform. In addition, we also included gene expression data that matched the DNA methylation data from the GEO series GSE85217 (N = 763) profiled using Affymetrix Human Gene 1.1 ST Array.

**TABLE 1:** Overview of datasets used in the current study from GEO Series: Testing, training, validation, and integration Dataset. Age and sex were predicted for datasets with missing metadata information.

Dataset	GEO accession	Total samples	Age (years) (mean ± SD)	Gender (% male)	Country	References
Training/Testing	GSE85212^a^	763	10.43 ± 9.43	65.65	Canada	Cavalli et al. (2017)
Integration	GSE85217, GSE85212	763	10.43 ± 9.43	65.65	Canada	Cavalli et al. (2017)
Validation	GSE130051	1390	5.78 ± 10.53	66.14	Europe, North America and Asia-Pacific	Sharma et al. (2019)
Validation	GSE90496	390	36.15 ± 6.27	60.26	Germany	Capper et al. (2018)
Validation	GSE54880^a^	276	8.27 ± 4.75	63.04	Germany	Hovestadt et al. (2013)
Validation	GSE109379	128	36.75 ± 6.84	60.47	Germany	Capper et al. (2018)
Validation	GSE75153	91	11.5 ± 18.39	59.78	Canada	-

^a^ Series with original metadata.

### 2.2 Methylation data preprocessing

We downloaded raw data files in “idat” format for all the aforementioned GEO datasets and assessed their quality using the minfi Bioconductor package (Aryee et al., 2014a). Subsequently, we conducted the following preprocessing procedure:

a) We assessed the signal quality using the detectionP function from the Bioconductor minfi package. We then calculated the p-values for each CpG probe across all samples. Probes with a *p*-value >0.05 in over 5% of samples were removed from subsequent analysis.
b) As all samples used in the current study were from the cerebellum, we used the preprocessQuantile function from the minfi package to normalize the data. We excluded CpG probes related to sex chromosomes and probes associated with single nucleotide polymorphisms (SNPs). On average, the total number of remaining probes was 420,000.
c) The methylation beta values ranging between 0 and 1 were calculated using the getBeta function from the Bioconductor minfi package. Briefly, such values were obtained based on the methylated and unmethylated probe intensities using formula M/(M + U + 100) (Bibikova et al., 2011); M and U stand for fully methylated and fully unmethylated intensities, respectively.
d) To deduce missing demographic information, including age and sex, we employed the methyAge algorithm and the predictedSex function from the Enmix (Xu et al., 2021) and minfi (Aryee et al., 2014) packages, respectively. This allowed us to create a summarized demographic view of the data types used in the current study.

### 2.3 Integration of DNA methylation and gene expression data using similarity network fusion (SNF)

In our study, we utilized the similarity network fusion (SNF) technique (Wang et al., 2014) proposed by Wang *et al.* to integrate the DNA methylation dataset with gene expression data and to further generate new labels. SNF allows for the identification of similarity networks, enabling the creation of the most appropriate labels for the methylation dataset using spectral clustering. To this end, we combined 763 samples from the methylation dataset (GSE85212) with the same number of samples from the gene expression dataset (GSE85217). The data integration was performed using the following parameters: 51 nearest neighbors, sigma = 0.85, and 120 iterations. As our study focused on medulloblastoma, which is characterized by the four subgroups, we set the cluster number to four and used the result of spectral clustering as the ground truth labels. We converted the cluster numbers into subgroups by comparing the sample number from the fused dataset and actual labels. Next, we evaluated the performance of the fused network by calculating the normalized mutual information (NMI) score, ranging from 0 to 1. An NMI score of 1 indicates that the fused network leads to the same labels as the actual labels, while a score of 0 indicates the opposite.

### 2.4 Feature selection

Feature selection is a critical step in machine learning, as it allows for the identification of the most relevant features, resulting in decreased prediction model error rates and computational time. In this study, we utilized a random forest model (RF) to train the top 5,000 most variable CpG probes obtained from Median Absolute Deviation (MAD) through the mad function in the stats package. To this end, we grew 300 trees using the RF model and determined the importance of each probe across all subgroups using the varImp function from the caret package.

### 2.5 Survival analysis

To evaluate the prognostic potential of prediction biomarkers, we conducted an overall survival analysis by adapting the MethSurv webtool pipeline (Modhukur et al., 2018; Modhukur, 2019). We utilized a multivariate Cox proportional hazards model to associate the methylation levels of each biomarker with patient survival using age, sex and MB subgroups as covariates. Patients were divided into high and low methylation groups based on a cut-off point such as the mean, median, or upper and lower quantiles. The specific cut-off values were determined based on models with high hazard ratios (HRs), maximizing the difference in survival outcomes between the groups. Next, we evaluated the goodness of fit of the Cox model using both the likelihood-ratio (LR) test and the Wald test.

### 2.6 Class imbalance correction

To overcome the challenge posed by imbalanced sample sizes for each MB subgroup in the methylome data, we implemented a technique called synthetic minority oversampling (SMOTE) (Chawla et al., 2002) using the DMwR package (Torgo, 2016). SMOTE generates synthetic samples by interpolating between existing minority class samples.

### 2.7 Data clustering

We utilized t-distributed stochastic neighbor embedding (t-SNE), a non-linear dimensionality reduction technique using the Rtsne package (Van Der Maaten and Hinton, 2008), to reduce the high-dimensional space to the most informative variables. The resulting cluster labels from the previous spectral clustering step were applied to identify four subgroups in our dataset, which were visualized in a three-dimensional (3D) plot using the rgl package (Adler et al., 2003). To explore the distribution of beta values, we used the ComplexHeatmap R package (Gu et al., 2016) to generate heatmaps.

### 2.8 AI-based models to classify MB subgroups

Our aim was to address the multiclassification challenge of accurately classifying medulloblastoma (MB) subgroups by leveraging the DNA methylation levels as a key feature. To do this, we used a diverse set of machine learning algorithms. The six algorithms employed were random forest (RF), naive Bayes (NB), K-nearest neighbor (KNN), support vector machine (SVM), extreme gradient boosting (XGB), and linear discriminant analysis (LDA). Furthermore, to capture the intricate nonlinear relationships, we incorporated an artificial neural network (ANN) model. Since the ensemble-based algorithms RF and XGB combine the predictions of multiple weak models to improve overall performance, we included those models in our study. On the other hand, NB operates as a probabilistic model, employing Bayes’ theorem to calculate the likelihood of class membership based on the independent features. KNN is classified as a nonparametric supervised learning algorithm, meaning that it does not make explicit assumptions about the underlying data distribution and defers computations until prediction. SVM can function either as a linear or as a nonlinear model, using a hyperplane or kernel trick to separate classes in the feature space. LDA is a linear model that projects data onto a lower-dimensional space to maximize class separation, aiding classification (Ray, 2019). The utilization of diverse machine learning algorithms in this classification conundrum enables a comprehensive evaluation of their efficacies, fostering heightened precision and resilience of the classification model. Additionally, ensemble methods (RF and XGB) can reduce variance and bias, while linear models (SVM and LDA) provide interpretability of the results (Sheth et al., 2022). Moreover, the ANN model is well known for its capability to learn complex nonlinear relationships between features. Unlike linear models, ANNs consist of interconnected nodes or neurons organized in layers, enabling them to capture intricate patterns and interactions in the data (Grossi and Buscema, 2007).

To train the abovementioned machine learning prediction models, we split the data into the training and test sets with a ratio of 0.8 for machine learning models using the sample. split function from the caTools package. Furthermore, we performed cross-validation in ten random folds (k = 10) using the createFolds function from the caret package (Kuhn, 2008).

The RF model was trained using the Random Forest package (Liaw and Wiener, 2002) with 300 trees and six as the maximum number of nodes. The SVM and NB models were trained using the e1071 package (Meyer, 2014), and a threshold of 0.8 was defined for NB to convert probabilities into subgroups. The KNN model was trained using the class package (Venables and Ripley, 2013) with three nearest neighbors, and the LDA model was trained using the lda function from the MASS package.

We implemented ANN models using the Keras package in R with TensorFlow 2.10 (Abadi et al., 2016). The data were split into training, testing, and validation sets with ratios of 0.6, 0.2, and 0.2, respectively. The ANN model had four layers: input, two hidden layers, and output, with neuron counts of 40, 30, 10, and 4. ‘Leaky ReLU’ activation was used for the first three layers, and softmax was used for the output layer.

To prevent overfitting, we applied regularization techniques, including dropout (50%, 40%, and 10% rates), L2 regularization on the second layer (regularizer_l2 = 0.009), and early stopping after five patients. The model was optimized using the categorical cross-entropy loss, stochastic gradient descent (SGD) optimizer, 200 epochs, batch size of 16, learning rate of 0.03, decay of 0.00006, momentum of 0.05, and Nesterov momentum.

To optimize the computational training time, we utilized the mclapply function from the parallel package to run the machine learning models in parallel on available CPUs. The training was performed on an Ubuntu machine equipped with an Intel Core i5-6200U processor and 16 GB RAM.

### 2.9 Performance evaluation

In our study, we evaluated the performance of each classification model using standard metrics, which included accuracy, sensitivity, specificity, precision, F1-score, and area under the curve (AUC) as described by similar studies (Le et al., 2017; Le et al., 2022). Briefly, the performance metrics were computed as follows:

$$Accuracy=\left(TP+TN\right)/\left(TP+TN+FP+FN\right)$$

$$Sensitivity=TP/\left(TP+FN\right)$$

$$Precision=TP/\left(TP+FP\right)$$

$$F1score=\left(2\times TP\right)/\left(2\times TP+FP+FN\right)$$

Here, true positives (TP), false positives (FP), true negatives (TN), and false negatives (FN) indicate whether the model predicted correctly or incorrectly. We also computed the AUC from the pROC package (Robin et al., 2011). The AUC score, presents the degree of separability between the classes.

### 2.10 Model visualization

To plot the training and testing results of a classifier, we designed a custom R script. Initially, the dataset was partitioned into a training set and a test set. Subsequently, principal component analysis (PCA) was conducted on the training and testing sets separately using the preProcess function from the caret package, enabling the extraction of two primary components that captured the most significant variability in the data. Following this, the training and test sets were transformed using the derived PCA outcomes. A grid structure was then constructed, encompassing values pertaining to the two principal components. Utilizing the trained classifier, labels are predicted for the grid set. Moreover, a color mapping scheme was employed to associate colors with the predicted and actual subgroups, enhancing the interpretability of the resulting plot.

### 2.11 Gene set enrichment analysis

To investigate the molecular function of the predicted CpG biomarkers and their relevance to the MB subgroups, we performed gene enrichment analysis. To annotate the CpGs with the genes, we utilized the minfi and IlluminaHumanMethylation450kanno.ilmn12.hg19 packages. The resulting genes were used as the input for the gprofiler2 package (Kolberg et al., 2020) to identify their gene ontology (GO) terms in the biological process (BP), KEGG, and Reactome pathways. To determine statistical significance, we used the false discovery rate (FDR) with a threshold of *p*-value <0.05.

### 2.12 Explaining the effect of each feature on the model output

To interpret the contribution of each identified biomarker to the MB subgroup prediction, we used the Shapley value, which is a local interpretation method in IML (Interpretable Machine Learning). Since the machine learning models employed in this study cannot directly elucidate the relationship between CpG probes and their target class, we employed the Shapley value to provide human-understandable explanations of the models’ results. The Shapley value is computed as the average marginal contribution of a CpG probe or gene beta value across all possible coalitions. For a single prediction of each MB subgroup, it randomly changed the value of each beta value from zero to the actual value of the sample and calculated the prediction for all patterns of changes due to the addition of each CpG. We used the iml package (Molnar, 2018) to calculate the Shapley values. To perform the Shapley analysis, we first trained an ANN model with all converted gene symbols from the functional enrichment step and the respective parameters as described in the iml package. Following the prediction on the training set, we used the prediction variable as input to the Shapley function to explain four samples of the training set belonging to each subgroup.

### 2.13 Network analysis

In this study, we utilized the igraph package (Csardi and Nepusz, 2006) to perform gene network analysis and investigate the relationship between the predicted genes. To identify clusters of genes that are highly correlated, we computed the Pearson correlation coefficient between each pair of genes and generated an adjacency matrix. We filtered out any edges that formed loops or had multiple connections, as well as edges with a Pearson correlation value less than or equal to 0.6 or genes with fewer than two adjacent edges. Additionally, we scaled the size of each gene according to its methylation values by a factor of 10 to enhance the readability of the network. We then utilized Prim’s algorithm to convert the graph adjacency object into a minimum spanning tree. Finally, we identified highly correlated gene clusters using a function called cluster_edge_betweenness.

## 3 Results

**FIGURE 1:**
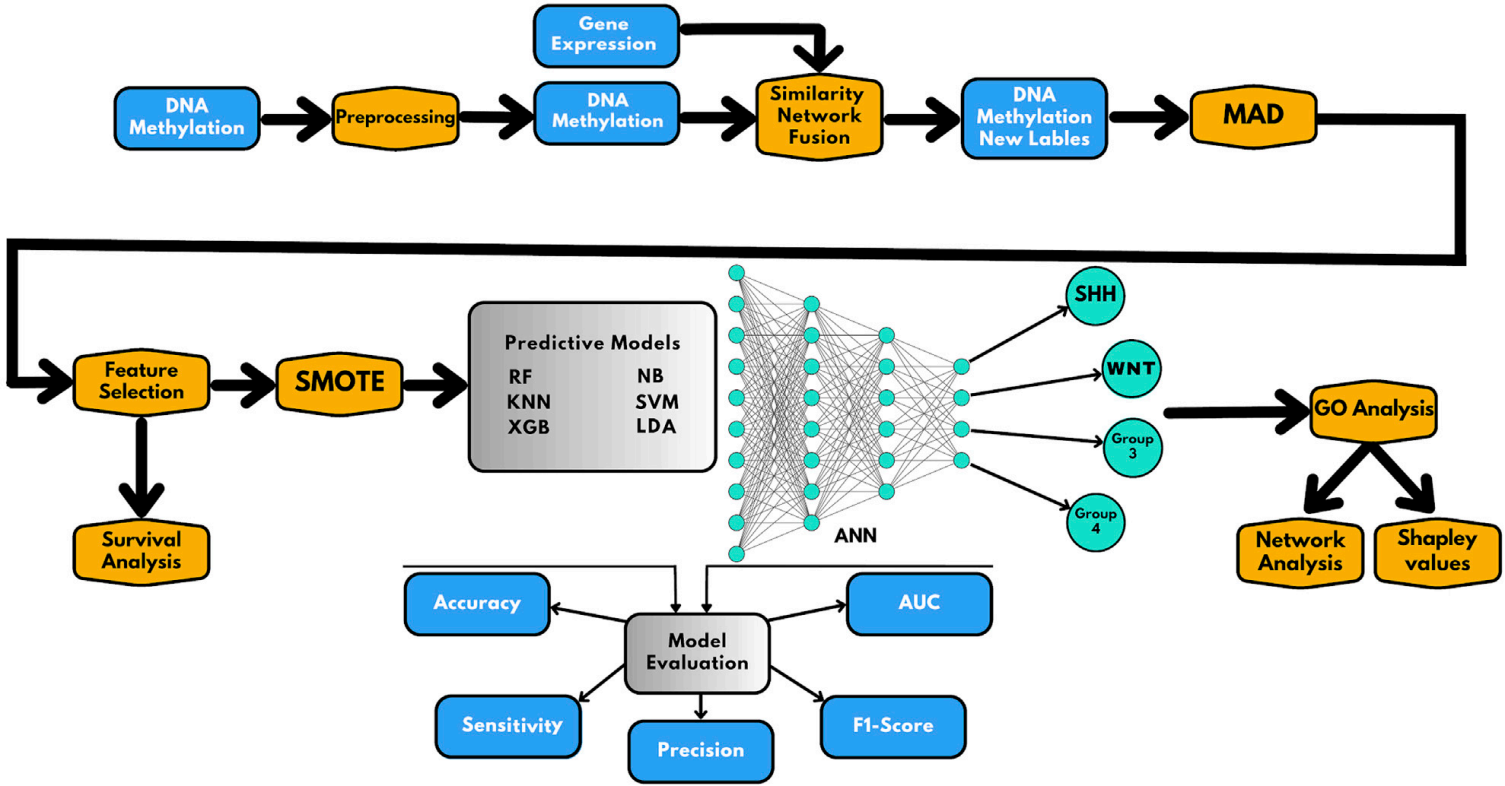
Schematic representation of the workflow presented in this study. The workflow includes the following steps: (1) preprocessing and integration of methylome and gene expression data; (2) similarity network fusion (SNF) to establish the new class labels; (3) MAD analysis for selecting informative prediction biomarkers and feature selection and survival analysis; (4) applying SMOTE for correction of class imbalance and construction of AI-based prediction models; (5) evaluation of the prediction models; and (6) Gene Ontology (GO) analysis for functional annotation of the prediction genes, where gene network analysis and Shapley values are used to understand the classifier results.

In this study, we used a combination of data integration and AI-based techniques to effectively classify subgroups of medulloblastoma. The methodology used in this study is presented in Figure 1 and involves the following six main steps:

(i) Collection of data from Gene Expression Omnibus (GEO), followed by pre-processing and processing steps;
(ii) Implementation of similarity network fusion (SNF) to establish new class labels by integrating DNA methylation and gene expression data;
(iii) Median Absolute Deviation (MAD) analysis was applied to select informative prediction biomarkers, followed by random forest (RF) analysis for feature selection. Furthermore, survival analysis was performed based on the prediction biomarkers.
(iv) Construction of AI-based prediction models following Synthetic Minority Oversampling Technique (SMOTE) application;
(v) Evaluation of the models using multiple parameters, including accuracy, sensitivity, precision, AUC, and F1-score;
(vi) Gene Ontology (GO) analysis was used to functionally annotate the selected genes.

We further conducted gene network analysis and interpreted the classifier decision by utilizing Shapley values. The subsequent sections provide detailed results from each of the steps mentioned above.

### 3.1 Integration of gene expression and methylation data through similarity network fusion

In this study, using similarity network fusion (SNF), we identified four distinct clusters in both the gene expression and methylation datasets (Supplementary Figures S1A, B). We then fused the resulting networks to obtain a comprehensive view of the data (Supplementary Figures S1C). The spectral clustering results on the fused network revealed two clusters (belonging to groups 3 and 4; Supplementary Figures S1C) with slightly different samples from the actual clusters (GSE85212) with a high NMI score of 0.926. Using the class labels obtained from SNF and implementing SMOTE, we addressed class imbalance, particularly in the minority subgroup (WNT = 70), by increasing the number of WNT samples to 210, resulting in a total of 910 samples (Supplementary Figures S2). Additionally, for the selection of the top 399 probes as features for prediction, we employed the random forest feature selection method among the 5,000 most variable probes identified using the median absolute deviation (MAD) method. This two-step process allowed us to first identify the 5,000 most variable probes based on MAD and then further reduce them to the top 399 probes using random forest feature selection (Supplementary Data S1).

**FIGURE 2:**
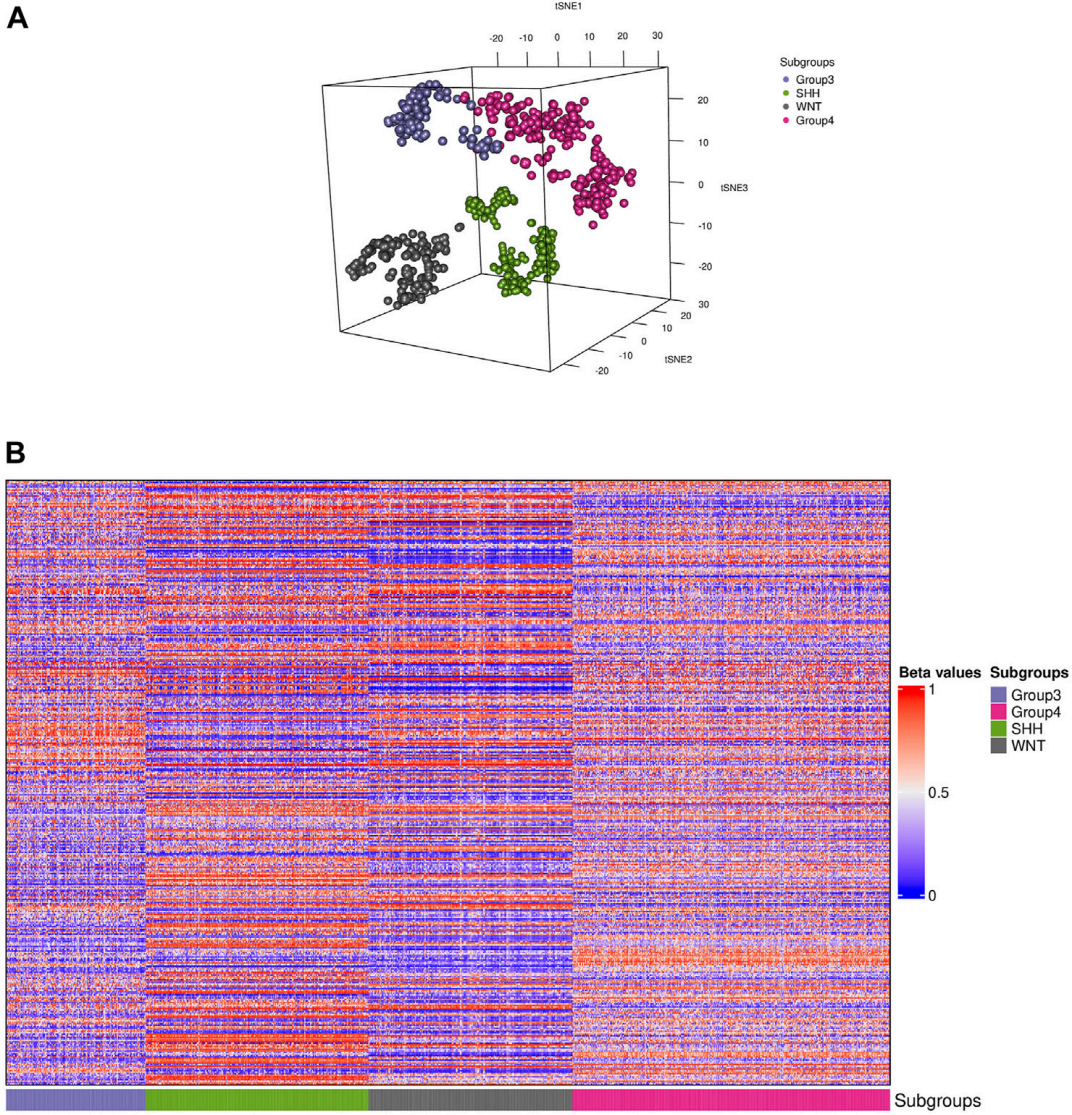
Visualization of the training data. **(A)** Distributed stochastic neighbor embedding (t-SNE) plot shows the presence of four distinct subgroups (colored dots) of medulloblastoma in the dataset. **(B)** A heatmap representation of the 910 samples depicting each subgroup is shown. The colors in the heatmap represent the levels of DNA methylation, with red indicating higher methylation levels and blue indicating lower methylation levels. The CpG biomarkers revealed a unique methylation pattern in groups 3 and 4, while the WNT and SHH subgroups displayed a distinct pattern.

The t-SNE visualization revealed (Figure 2A) only a minor overlap between groups 3 and 4; additionally, only one sample from the WNT cluster appeared in the SHH subgroup. Furthermore, we generated a heatmap of the CpG biomarkers to examine the distribution of methylation beta values across all subgroups (Figure 2B), in which a distinct methylation pattern among subgroups is notable.

### 3.2 Performance evaluation of the prediction models for medulloblastoma subgroup classification based on DNA methylation profiles

In our study, we employed six robust machine-learning algorithms, namely, SVM, KNN, NB, RF, XGB, and LDA, along with an artificial neural network, to predict medulloblastoma subgroups based on DNA methylation samples using 399 predictive biomarkers. As a result of the fusion process, a subset of samples (n = 16) had their labels switched (Supplementary Table S1). These new labels predominantly belonged to the Group 3 and Group 4 subgroups, accounting for 14 out of the 16 samples. These switched labels were utilized specifically for training the model. However, during the validation process, the confusion matrices were constructed based on the original labels from validation sets and predicted labels. For testing and training, we utilized the dataset from GSE85212, while multiple datasets were used for validation. Detailed information regarding the testing/training and validation datasets can be found in Table 1.

The overall performance of the classifiers based on the validation set (GSE90496) is presented in Table 2. Briefly, the ANN model achieved the highest accuracy of 99.25%, followed by SVM with 99.50% accuracy. However, the KNN, NB, RF, XGB, and LDA models also achieved high accuracy ranging from 97.80% to 99.35%.

**TABLE 2:** Overall performance metrics for each model using GSE90496 as a validation set.

Model	Accuracy	Precision	Sensitivity	F1.Score	Specificity	AUC
RF	0.9935 ± 0.005	0.98675 ± 0.013	0.988 ± 0.01	0.9875 ± 0.011	0.9955 ± 0.004	0.98 ± 0
SVM	0.995 ± 0.004	0.98875 ± 0.013	0.99125 ± 0.008	0.98975 ± 0.009	0.9965 ± 0.003	0.986 ± 0
XGB	0.9895 ± 0.005	0.979 ± 0.02	0.96875 ± 0.023	0.97325 ± 0.014	0.993 ± 0.005	0.973 ± 0
NB	0.9935 ± 0.005	0.98575 ± 0.017	0.9895 ± 0.01	0.9875 ± 0.011	0.99575 ± 0.004	0.983 ± 0
LDA	0.978 ± 0.017	0.95875 ± 0.032	0.95625 ± 0.04	0.9575 ± 0.036	0.9845 ± 0.012	0.928 ± 0
KNN	0.9885 ± 0.009	0.97775 ± 0.019	0.9765 ± 0.022	0.97725 ± 0.02	0.99175 ± 0.007	0.961 ± 0
ANN	0.9925 ± 0.078	0.98475 ± 0.17	0.98475 ± 0.17	0.98475 ± 0.17	0.9945 ± 0.058	0.995 ± 0

**FIGURE 3:**
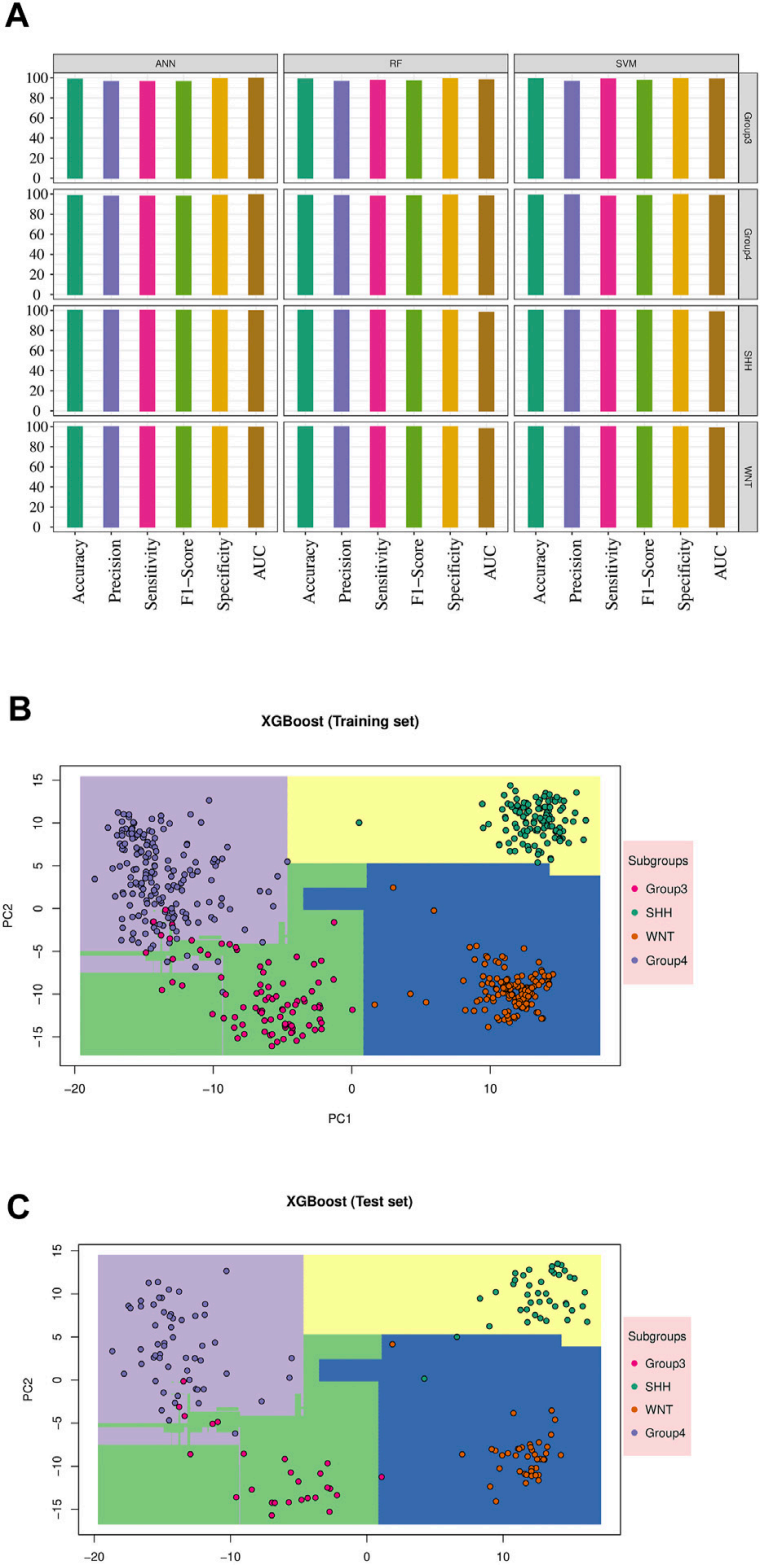
Machine learning model’s performance in predicting medulloblastoma subgroups: The result for validation cohort GSE90496 and XGBoost model results. **(A)** The performance of the top three models’ on the validation cohort GSE90496 (n = 390) is displayed (see Supplementary Figure S3 for other models). The *X*-axis represents various metrics, including accuracy, precision, sensitivity, F1-Score, specificity, and AUC. Each metric is represented by a bar plot using different colors, indicating the corresponding percentage. These metrics were calculated separately for each subgroup, highlighting the accurate classification of WNT and SHH subgroups, as well as some misclassifications within Groups 3 and 4. **(B)** The training result for the XGBoost model is shown. The distinct decision boundaries for each subgroup are denoted by various colors. **(C)** The testing result for the XGBoost model performance is displayed. Similar to the training results, distinct decision boundaries are depicted for each subgroup using different colors.

Since the focus of our study was the classification of MB subgroups, we evaluated the performance of each model, considering the different MB subgroups, across multiple validation datasets. Notably, all tested classifiers exhibited exceptional performance on the GSE90496 validation set, exceeding 0.92 in accuracy, precision, sensitivity, F1-Score, specificity, and AUC (Table 3; Figure 3A; Supplementary Figure S3). We specifically monitored the performance of the prediction models on the challenging Group 3 and Group 4 MB subgroups. The SVM, RF, and ANN models achieved excellent performance, with accuracy, precision, sensitivity, F1-Score, specificity, and AUC exceeding 0.96 (Table 3; Supplementary Table S2). Other models, including KNN, NB, LDA, and XGB, also demonstrated comparable performance, with accuracy, precision, sensitivity, F1-Score, specificity, and AUC ranging from 0.88 to 0.99 (Table 3; Supplementary Table S2; Supplementary Figure S3).

**TABLE 3:** Performance metrics of each model for MB subgroup classification using GSE90496 as a validation set.

Subgroup	Accuracy	Precision	Sensitivity	F1-score	Specificity		AUC	Model
Group3	0.987	0.962	0.974	0.968	0.99		0.98	RF
Group4	0.987	0.985	0.978	0.982	0.992		0.98	
SHH	1	1	1	1	1		0.98	
WNT	1	1	1	1	1		0.98	
Group3	0.99	0.962	0.987	0.974	0.99		0.986	SVM
Group4	0.99	0.993	0.978	0.985	0.996		0.986	
SHH	1	1	1	1	1		0.986	
WNT	1	1	1	1	1		0.986	
Group3	0.982	0.938	0.974	0.955	0.984		0.973	XGB
Group4	0.987	0.985	0.978	0.982	0.992		0.973	
SHH	0.997	0.993	1	0.996	0.996		0.973	
WNT	0.992	1	0.923	0.96	1		0.973	
Group3	0.987	0.95	0.987	0.968	0.987		0.983	NB
Group4	0.987	0.993	0.971	0.982	0.996		0.983	
SHH	1	1	1	1	1		0.983	
WNT	1	1	1	1	1		0.983	
Group3	0.959	0.907	0.883	0.895	0.978		0.928	LDA
Group4	0.956	0.935	0.942	0.939	0.964		0.928	
SHH	0.997	0.993	1	0.996	0.996		0.928	
WNT	1	1	1	1	1		0.928	
Group3	0.977	0.947	0.935	0.941	0.987		0.961	KNN
Group4	0.977	0.964	0.971	0.968	0.98		0.961	
SHH	1	1	1	1	1		0.961	
WNT	1	1	1	1	1		0.961	
Group3	0.985	0.961	0.961	0.961	0.99		0.995	ANN
Group4	0.985	0.978	0.978	0.978	0.988		0.995	
SHH	1	1	1	1	1		0.995	
WNT	1	1	1	1	1		0.995	

Furthermore, we visualized the ability of the classifiers based on the training and test sets, as shown in Figures 3B,C, using Principal Component Analysis (PCA) based on XGB as the reference model. The PCA plot revealed a clear separation between MB subgroups**.** Thus, the classifiers successfully captured the underlying variability and discriminating features among the different MB subgroups.

Across the different validation sets, our models consistently displayed higher performance. For example, on the GSE130051 dataset, the NB model emerged as a top-performing classifier with accuracy exceeding 0.96, while other models achieved accuracy ranging from 0.91 to 0.95 (Supplementary Tables S3, S4). The ANN model demonstrated robust performance on the GSE54880 dataset, achieving an accuracy of 0.97 with minimal misclassifications (Supplementary Tables S5, S6). On the GSE109379 dataset, the ANN and RF models performed exceptionally well, achieving accuracy above 0.97, while the SVM, XGBoost, and KNN models also exhibited favorable performance, albeit with slightly lower precision and sensitivity for Groups 3 and 4 (Supplementary Tables S7, S8). Finally, for the GSE75153 dataset, all models performed comparably well, with accuracy above 0.97 (Supplementary Tables S9, S10).

In summary, our analysis revealed slight variability in the performance of different prediction models across a diverse range of validation sets, with an average accuracy exceeding 0.96.

### 3.3 Biological and clinical significance of the prediction biomarkers

**FIGURE 4:**
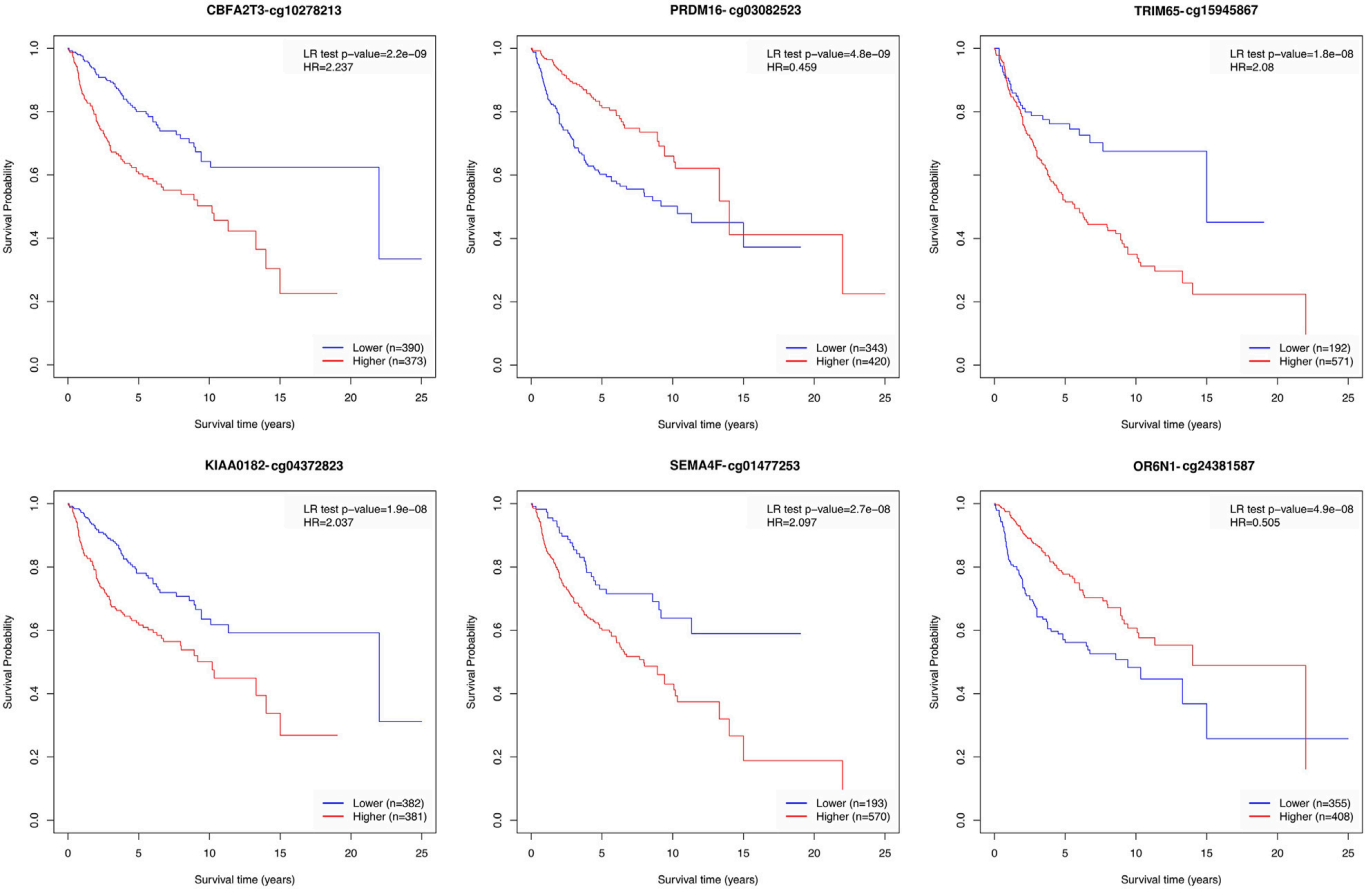
Kaplan–Meier plots depicting the effect of the top six prediction biomarkers (log-rank test <0.05). Methylation groups are dichotomized by higher and lower methylation groups based on a cut-off point such as the mean, median, or upper and lower quantiles. The *X*-axis denotes survival time in years, and the *Y*-axis denotes the probability of patient survival.

We performed an overall survival analysis on 399 prediction biomarkers after adjusting for the covariates age, sex and sugroups using the methodology adapted from MethSurv (Modhukur et al., 2018; Modhukur, 2019). We found that all 399 prediction biomarkers showed a significant association with patient survival (log rank test *p*-value <0.05). The top biomarkers with the lowest *p* values included *CBFA2T3*, *PRDM16*, *TRIM65*, *KIAA0182*, *SEMA4F*, *OR6N1*, *RPTOR*, *KIAA0415*, *SAG*, and *TTC15* (Figure 4; Supplementary Figure S4, and Supplementary Data S2).

To further gain biological insights into the prediction biomarkers, we performed functional enrichment analysis. We annotated each probe with its gene symbol and excluded CpGs without gene annotations. For CpGs with duplicated gene names, we calculated the median value. The latter resulted in a total of 239 unique gene symbols, which were used as input for gprofiler2 (Peterson et al., 2020). Our analysis identified the 20 most significant biological processes (adjusted *p*-value <0.05) in which the selected genes were enriched (Supplementary Figure S5). Some of these biological processes included nervous system development, neurogenesis, neuron projection development, and differentiation. To evaluate the effectiveness of the enriched genes, we employed a neural network as our optimal model to analyze genes associated with the top 20 biological processes. The neural network consisted of five layers with 50, 30, 20, 10, and 4 neurons and a learning rate of 0.03. We trained each gene set ten times and computed the average performance results. Although all models produced similar outcomes with AUC scores above 0.9, the nervous system development process consisting of 49 genes had the highest mean AUC score of 0.995 (Supplementary Figure S6; Supplementary Table S11).

### 3.4 Explaining feature effects on model output through Shapley values

**FIGURE 5:**
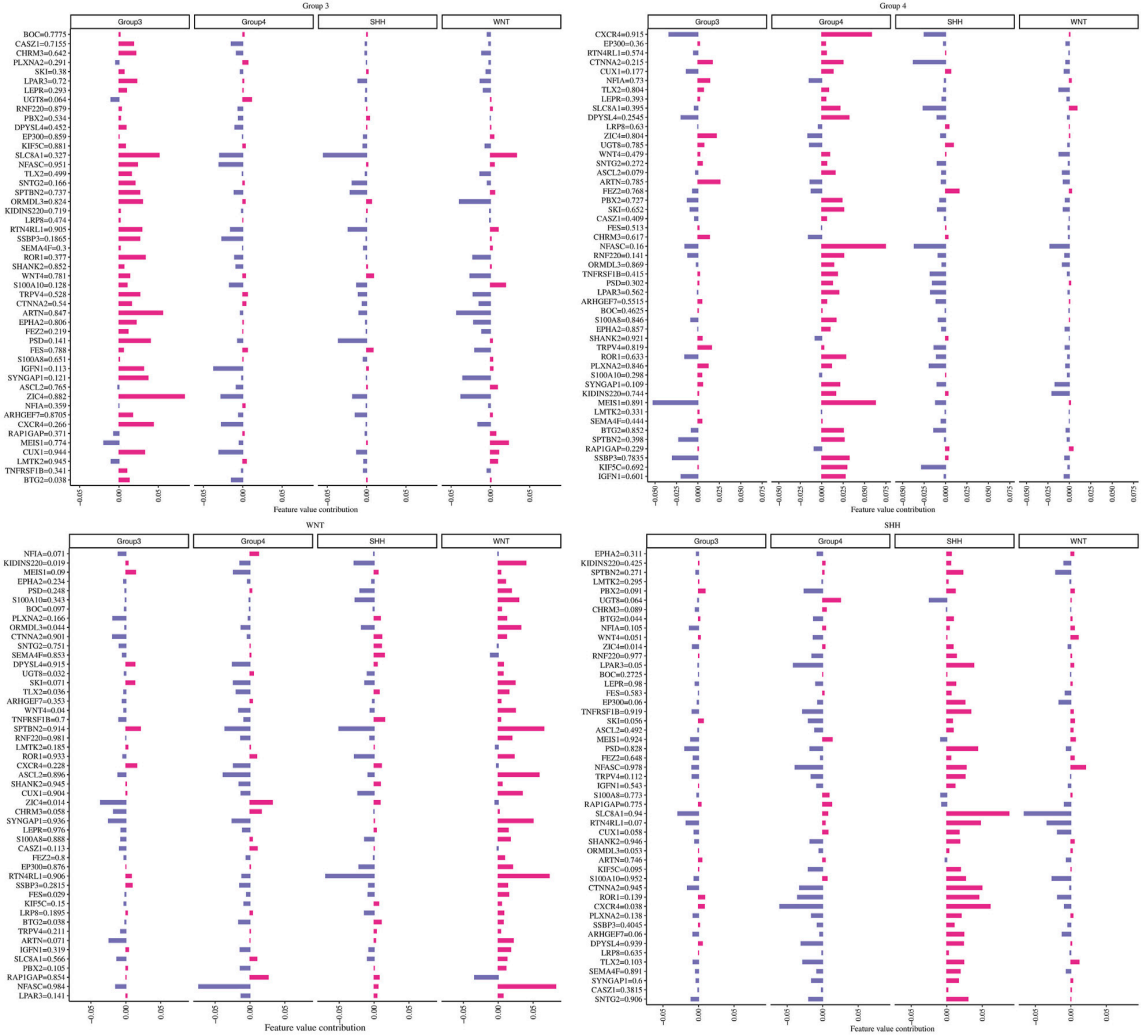
The contribution of each gene in predicting every MB subgroup. Each plot title corresponds to an associated group where we computed Shapley values. The *Y*-axis represents genes with their beta values, while the *X*-axis demonstrates the genes’ contribution to the ANN model based on the phi value.

To investigate the individual impact of the prediction genes (N = 49) on the model performance, we computed the Shapley values for the trained neural network model. Each gene with its corresponding beta values and their contribution in terms of Shapley values on the ANN model across different subgroups are shown in Figure 5. Briefly, maroon color indicates a positive effect, and blue denotes an adverse effect.

For example, we found that *ZIC4*’s hypermethylation state (beta value = 0.882) has a highly positive impact on the model’s ability to predict Group 3 but has a negative effect on the WNT subgroup. At the same time, *ZIC4* has a low negative impact on the model’s ability to forecast SHH and Group 4 subgroups. Additionally, we identified other genes, such as *ARTN* and *SLC8A1*, which have a positive contribution to the model’s ability to predict Group 3, with beta values equal to 0.847 and 0.327, respectively.

Furthermore, we observed that higher methylation levels of the *CXCR4* and *MEIS1* genes and lower methylation levels of *NFASC* had a positive impact on the ANN model’s ability to predict the Group 4 subgroup. In the WNT subgroup, *ASCL2*, *SYNGAP1*, *RTNR4L*, and *NFASC* gene hypermethylation status, as well as *KIDINS220* and *S100A10* gene hypomethylation, had a highly positive impact on prediction. For the SHH subgroup, we found that higher methylation levels of *SLC8A1* and lower methylation levels of *ROR1*, *CXCR4*, and *RTN4RL1* had a high contribution to the prediction.

### 3.5 Network analysis

**FIGURE 6:**
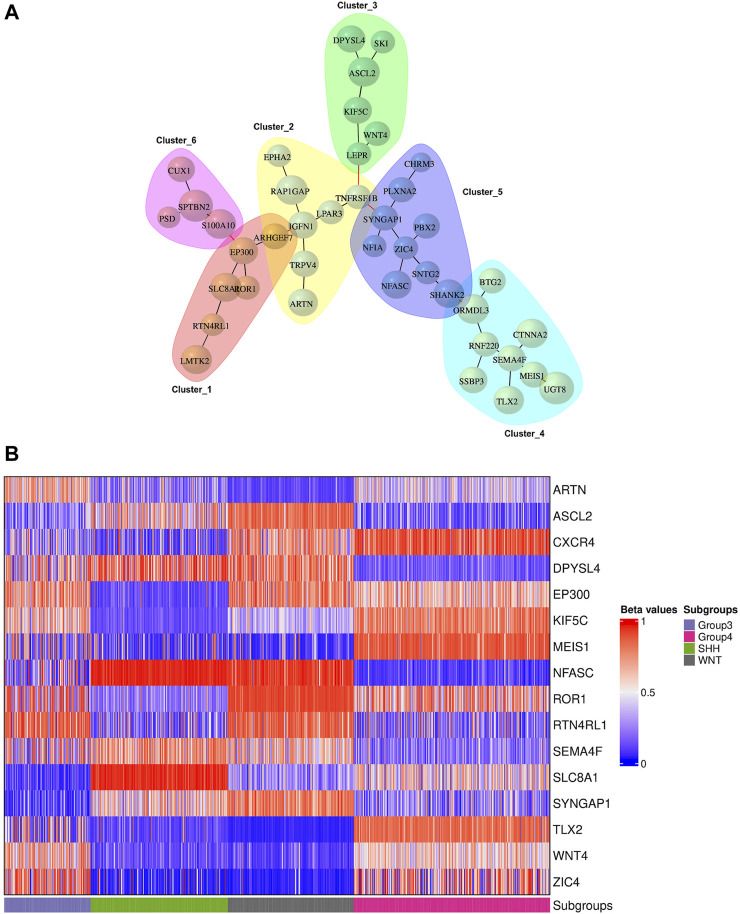
Gene network analysis and heatmap plot of the beta values associated with the prognostic genes. **(A)** Gene network representation of 41 out of 49 genes enriched in the nervous system development process, showing the correlation between genes belonging to six distinct clusters. The size of the vertices represents the beta values of the genes with a Pearson correlation coefficient above 0.6. **(B)** Methylation statuses (hypo in blue, hyper in red) of significant genes for the precise prediction of MB subgroups.

**FIGURE 7:**
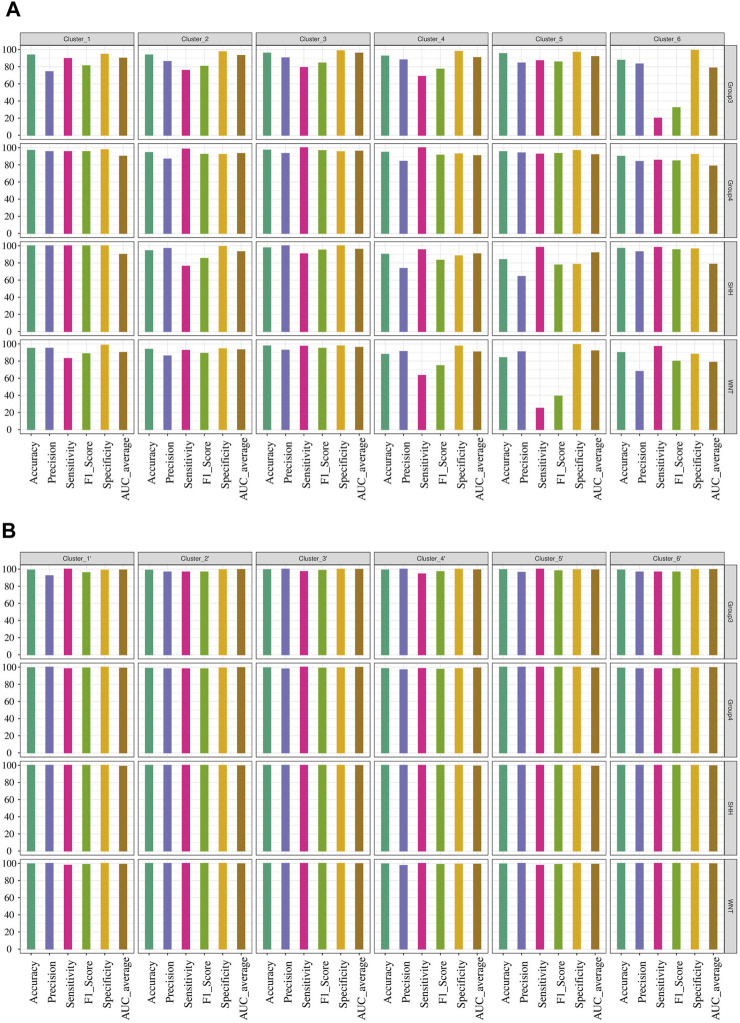
Performance evaluation of ANN models for predicting MB subgroups. **(A)** Prediction outcomes of MB subgroups using genes within each cluster derived from the network analysis. **(B)** Performance improvement of the ANN model by including additional genes in the existing gene list within each cluster, resulting in the creation of a new cluster designated by the prime symbol.

We conducted network analysis using the methylation beta values of 49 genes enriched in the nervous system development process identified based on the functional enrichment analysis (Supplementary Figure S5; Supplementary Table S11). The resulting network revealed 41 genes with a Pearson correlation coefficient greater than 0.6, distributed among six distinct clusters (Figure 6A). To evaluate the classification ability of each cluster’s genes, we trained artificial neural network (ANN) models for each cluster. However, upon assessing the performance of the individual models on the test data (Figure 7A), we observed that some models exhibited poor performance for certain subgroups. To address this limitation, we devised a unique strategy to enhance the model’s performance. Specifically, we incorporated genes from other clusters into each model until we achieved improved performance (Figure 7B). This iterative process allowed us to leverage the collective predictive power of multiple gene clusters, ultimately leading to enhanced classification accuracy. The performance of each model on the test data is shown in Figure 7A, where all models except for cluster 3 exhibited poor performance. To improve the model’s performance, we gradually added genes from other clusters to each model until the performance improved (Figure 7B). Accordingly, we confirmed the significance of *ARTN* and *WNT4* for Group 3 and WNT subgroups, respectively. These genes suggest possible associations with their respective subgroups, highlighting their importance in driving molecular characteristics and prognostic outcomes. Building upon these findings, we integrated *ARTN*, *WNT4*, *EP300* and *ROR1* into the gene list of cluster 4, resulting in improved performance for cluster 1.

Furthermore, by adapting a similar procedure, we intended to improve cluster 5, which initially exhibited the lowest performance. To achieve this, we incorporated the additional genes *RTN4RL1*, *TLX2*, *ARTN*, *WNT4*, *EP300*, and *ROR1* into the existing gene list from cluster 5. Additionally, cluster 6 was improved by using the same gene list as cluster 5. However, for cluster 3, we included *SEMA4F, SLC8A1, CXCR4, SYNGAP1, NFASC,* and *MEIS1* in the existing list of significant genes, thereby improving its predictive power.

Figure 6B displays the beta values associated with the predicted prognostic genes. Furthermore, Table 4 provides a comprehensive list of these significant genes, highlighting their functional annotations and their relevance to each molecular subgroup.

**TABLE 4:** Predicted key prognostic genes associated with molecular subgroups of medulloblastoma.

Gene name	SHH	WNT	Group 3	Group 4	Function
*EP300*	✓				Histone acetyltransferase; regulates cell proliferation and differentiation
*CXCR4*	✓		✓	✓	Chemokine receptor with high expression in breast cancer cells
*WNT4*		✓			Involved in oncogenesis and developmental processes, such as embryogenesis
*ZIC4*			✓		Transcription factor; involved in cerebellum development
*MEIS1*				✓	Plays a crucial role in normal development
*SLC8A1*	✓		✓	✓	Sodium-calcium exchanger
*ASCL2*		✓			Transcription factor; involved in the determination of the neuronal precursors in the peripheral nervous system and the central nervous system (CNS)
*NFASC*		✓		✓	Cell adhesion
*KIF5C*	✓			✓	Transport of cargo in CNS
*SYNGAP1*		✓	✓	✓	Ras GTPase; regulates synaptic plasticity and neuronal homeostasis
*SEMA4F*	✓				Neural development
*ROR1*	✓	✓	✓	✓	Neurite growth in CNS
*DPYSL4*	✓			✓	Development of the enteric nervous system (in mouse)
*ARTN*			✓		Supports the survival of several peripheral neuron populations and at least one population of dopaminergic CNS neurons
*RTN4RL1*	✓	✓	✓		Negative regulation of axon regeneration
*TLX2*			✓	✓	Transcription factor; involved in development of the enteric nervous system

## 4 Discussion

Accurate classification of molecular subgroups in medulloblastoma (MB) is vital for initiating appropriate treatment plans. In our study, we utilized a comprehensive approach integrating data and AI-based methods and utilized synthetic sample generation using SMOTE to address limited data and maintain class balance. Our developed prediction framework, MBMethPred, was designed explicitly for medulloblastoma subgroup classification using DNA methylation data. MBMethpred incorporates multiple AI models to enhance accuracy, processing speed, ease of use, and user-friendliness.

Compared to the molecular-based MB subgroup classification methods (Schwalbe et al., 2013; 2017; Korshunov et al., 2017; Capper et al., 2018; Gomez et al., 2018; Korshunov et al., 2019; Sharma et al., 2019; Rathi et al., 2020) (Supplementary Table S12), MBMethPred demonstrates several distinctive characteristics and advantages. Previous studies (Schwalbe et al., 2013; Korshunov et al., 2017; Schwalbe et al., 2017; Capper et al., 2018; Gomez et al., 2018; Korshunov et al., 2019; Sharma et al., 2019) employed a single classifier, in contrast to MBMethPred, which applies multiple classifiers. While MBMethPred achieves an AUC score above 0.99, the primary focus of Capper *et al.*'s (2018) study was the classification of central nervous system tumors, rather than focusing on medulloblastoma. Furthermore, it lacked an accuracy score specifically for medulloblastoma. Similarly, Sharma *et al.* exclusively concentrated on the classification of Groups 3 and 4 subgroups. Additionally, both Korshunov et al., 2017 and Korshunov et al. (2019) utilized smaller sample sizes (N = 239 and N = 78, respectively) compared to MBMethPred’s sample size of 910 samples. Likewise, Korshunov et al. (2019) solely focused on classifying the WNT subgroup. Moreover, Rathi et al. (2020) and Gomez et al. (2018) reported accuracies ranging between 85% and 100% using a single classifier, which is lower than the accuracy achieved by MBMethPred with multiple classifiers**.** In contrast, Attallah and Zaghlool (2022) utilized histopathology images and achieved 100% accuracy (Attallah and Zaghlool, 2022). However, there is limited availability of histopathological images and a lack of precision (Kim et al., 2022). This approach may restrict its widespread applicability. In this context, MBMethPred remains an accessible and valuable alternative for medulloblastoma subgroup classification complemented by its robust performance and comprehensive evaluation in comparison to the existing methods.

Our study comprehensively evaluated the models’ effectiveness in classifying MB subgroups using multiple validation datasets. Although slight variations were observed in the performance of prediction models across different datasets, the overall high performance observed in our study strengthens the reliability and generalizability of the models. Thus, incorporating multiple validation sets and prediction models is essential for robust evaluation of model reliability.

Gene-specific effects on model prediction were identified using Shapley values, offering insights into the contributions of specific genes to subgroup classification. Additionally, survival analysis identified significant associations between the identified biomarkers and survival outcomes in MB patients. Moreover, the biomarkers with significant survival outcomes correlated with previously reported oncogenes. For example, the CBFA complex, which includes *CBFA2T3* (Hendrikse et al., 2022; Gorini et al., 2023), is suggested to play a critical role in tumor development through its interactions with epigenetic modifiers, contributing to the pathogenesis of medulloblastoma*.* Similarly, the study by Menyhárt et al. (2019) demonstrated epigenetic changes in the *RPTOR* gene, along with other identified biomarkers, in classifying non-WNT/non-SHH medulloblastomas*.* These findings suggest that the identified biomarkers hold the potential for predicting patient prognosis and guiding treatment decisions.

Our functional enrichment analysis highlighted the association between the model performance and biological relevance. For instance, *EP300* encodes a histone acetyltransferase protein that activates the expression of genes critical for the development and progression of medulloblastoma (Northcott et al., 2017). *CXCR4* has been suggested to be the oncogenic driver of MB (Amarante et al., 2018). In addition, *SYNGAP1* is a GTPase-activating protein that is known to cause cognitive deficits by inducing alterations in glutamatergic neurotransmission (Berryer et al., 2016). Finally, *WNT4* is a member of the Wnt signaling pathway and has been associated with the pathogenesis of WNT and SHH subgroups (Taylor et al., 2012). Thus, the functional insights gained from our study may contribute to identifying potential therapeutic targets for each medulloblastoma subgroup.

Finally, network analysis considered correlations among genes enriched in nervous system development and identified distinct clusters with potential relevance to medulloblastoma. Moreover, training a separate artificial neural network model for each cluster improved the classification accuracy by gradually incorporating genes from different clusters. Thus, our integrative approach enhances the understanding of the complex molecular heterogeneity underlying medulloblastoma and provides a basis for further research.

It is important to acknowledge some limitations of our study. Although we utilized gene expression profiles for data integration and further implemented SNF to define the new labels, our prediction models exclusively rely on the DNA methylation datasets. However, it is worth highlighting that the availability and accessibility of additional datasets, especially those including diverse patient populations, are currently limited, potentially impacting the generalizability of our findings. Therefore, further research in this direction is highly warranted to explore the clinical applicability of our study.

In conclusion, we developed a robust classifier for medulloblastoma subgroup classification. Moreover, our functional enrichment analysis offers valuable insights into the molecular pathogenesis of medulloblastoma. Survival analysis enables the evaluation of prognostic relevance for individual biomarkers. By identifying key genes in medulloblastoma subgroup classification and their functional relevance, our study provides insights into disease stratification. While our approach has the potential to be adapted for subgroup prediction in other cancer types, it requires careful validation and adaptation to specific datasets to ensure its reliability. Despite the underlying limitations, our findings contribute to the advancement of medulloblastoma research, with the potential to improve patient outcomes.

## Data Availability

Publicly available datasets were analyzed in this study. This data can be found here: https://www.ncbi.nlm.nih.gov/geo/. The package developed in this study is available from https://cran.r-project.org/web/packages/MBMethPred/index.html.
