# Supplementary material for: MBMethPred: a computational framework for the accurate classification of childhood medulloblastoma subgroups using data integration and AI-based approaches
Source: Front Genet. 2023 Sep 7;14:1233657. doi: 10.3389/fgene.2023.1233657 (PMC10513500; doi:10.3389/fgene.2023.1233657)
Supplement: Supplementary file 2 [file Table1.docx]

*Supplementary Information*

**MBMethPred: A Computational Framework for Accurate Classification of Childhood Medulloblastoma Subgroups using Data Integration and AI-Based Approaches**

**Edris Sharif Rahmani^1^, Ankita Lawarde^1,2^, Prakash Lingasamy^1^, Sergio Vela Moreno ^1,2,^ Andres Salumets^1,2,3^, Vijayachitra Modhukur^1,2*^**

^1^ Competence Centre on Health Technologies Tartu, Tartu, Estonia

^2^ Department of Obstetrics and Gynecology, Institute of Clinical Medicine, University of Tartu, Tartu, Estonia

^3^ Division of Obstetrics and Gynecology, Department of Clinical Science, Intervention and Technology, Karolinska Institute and Karolinska University Hospital, Stockholm, Sweden

*** Correspondence:**

Vijayachitra Modhukur

[modhukur@ut.ee](mailto:modhukur@ut.ee)

Keywords: Childhood Medulloblastoma, Subgroup Classification, DNA Methylation, Machine Learning, Neural Networks, Deep Learning, Gene Expression, Functional Enrichment Analysis, Shapley Values, Gene Network Analysis, WNT, SHH, Group 3, Group 4,


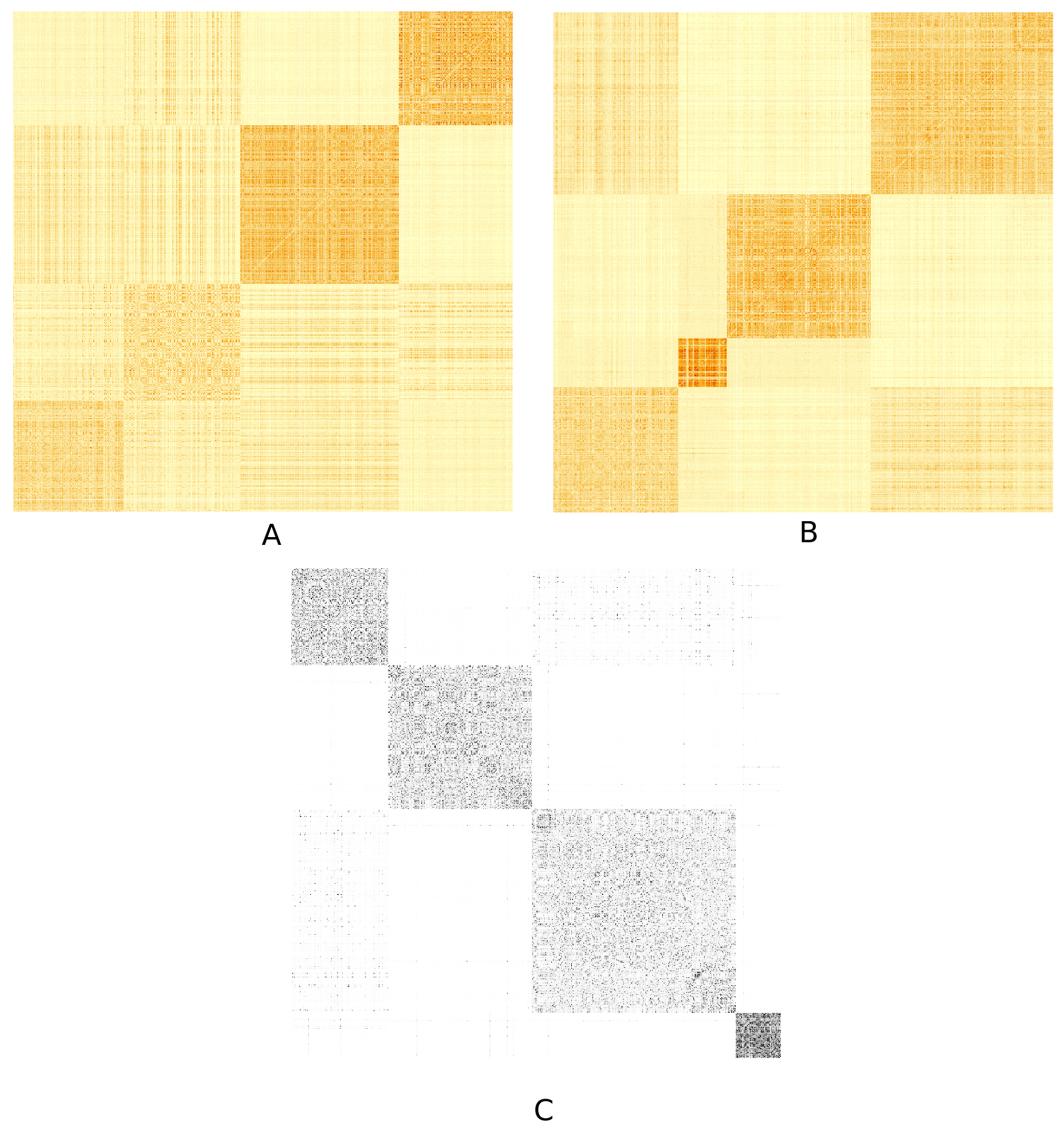


**Supplementary Figure S1.** Illustration of Similarity Network Fusion. (A) The distance matrix derived from the gene expression data reveals four distinct clusters, and (B) the corresponding distance matrix obtained from the methylation data also shows four distinct clusters. The integration of these two matrices is depicted in (C), resulting in a separate network with four distinct clusters.


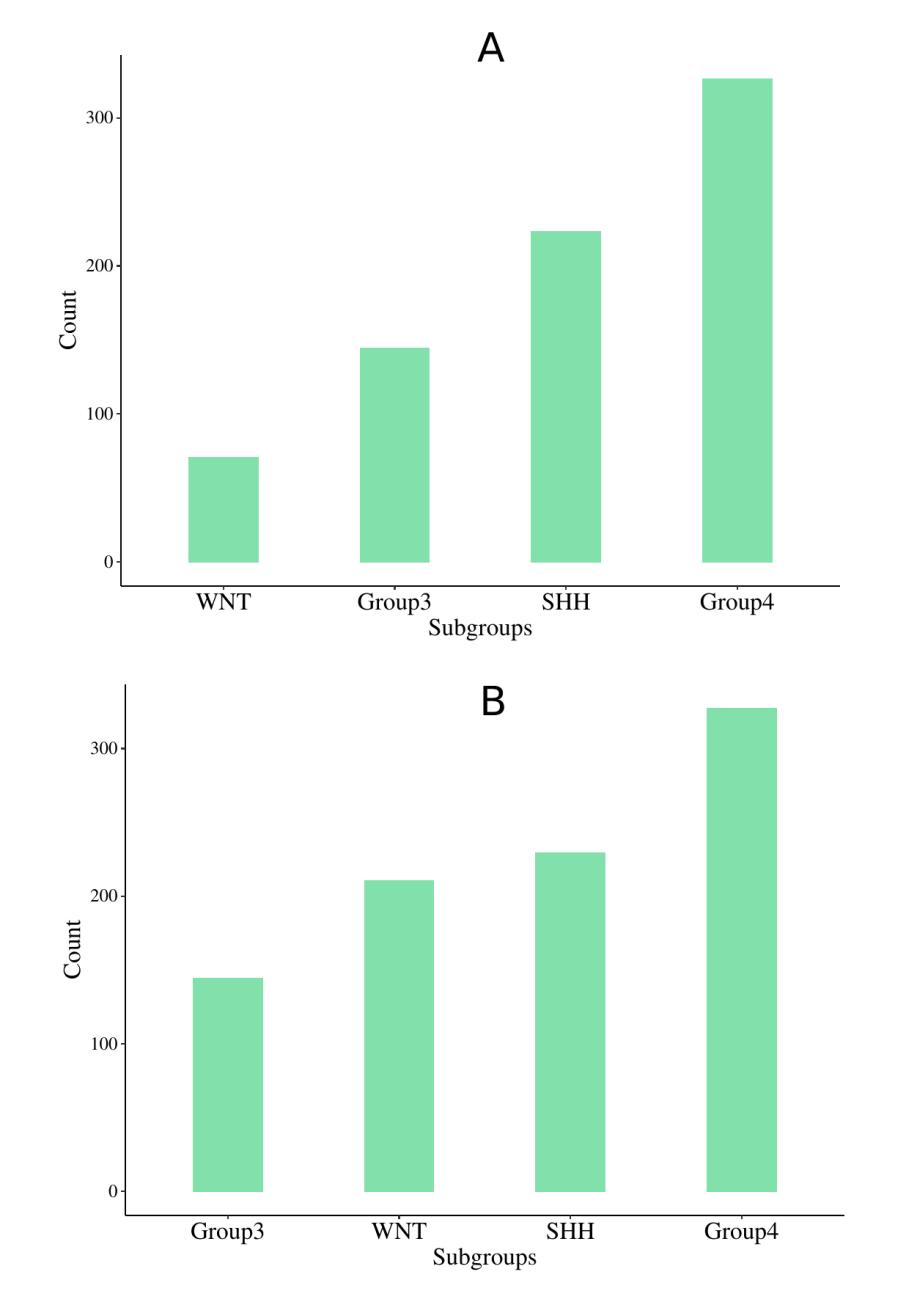


**Supplementary Figure S2.** The outcome of the Synthetic Minority Over-sampling Technique (SMOTE) applied to address the class imbalance. (A) Bar plot illustrating the significantly lower sample count of the WNT subgroup, which represents the minority class. (B) Bar plot illustrating the oversampling of the WNT subgroup, resulting in a more balanced dataset with an increased sample count.


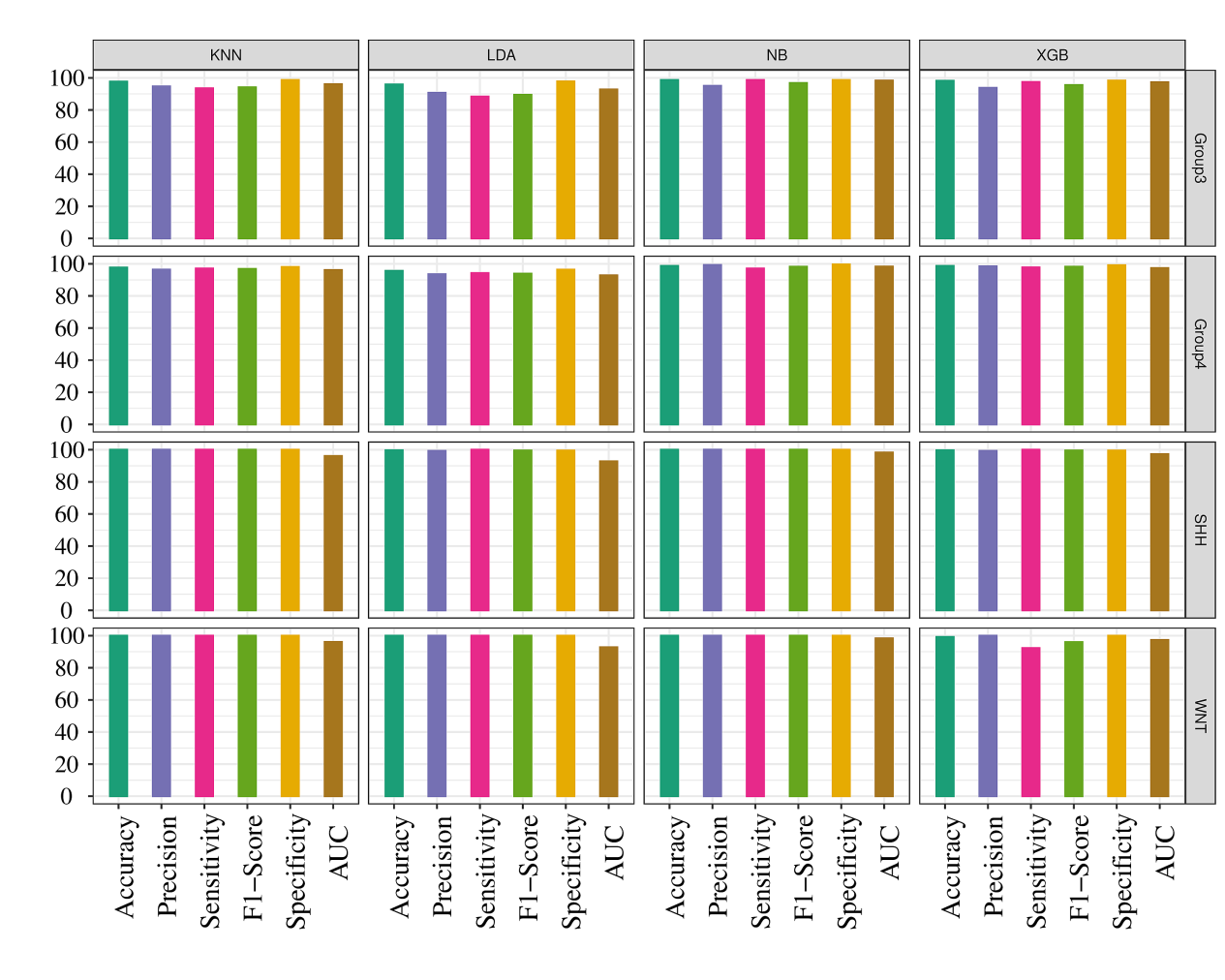


**Supplementary Figure S3.** The performance of the KNN, LDA, RF, and XGB (XGBoost) models on the validation cohort GSE90496 (n=390) is displayed. The X-axis represents various metrics, including accuracy, precision, sensitivity, F1-Score, specificity, and AUC. Each metric is represented by a bar plot using different colors, indicating the corresponding percentage. These metrics were calculated separately for each subgroup, highlighting the accurate classification of WNT and SHH subgroups, as well as some misclassifications within Groups 3 and 4.


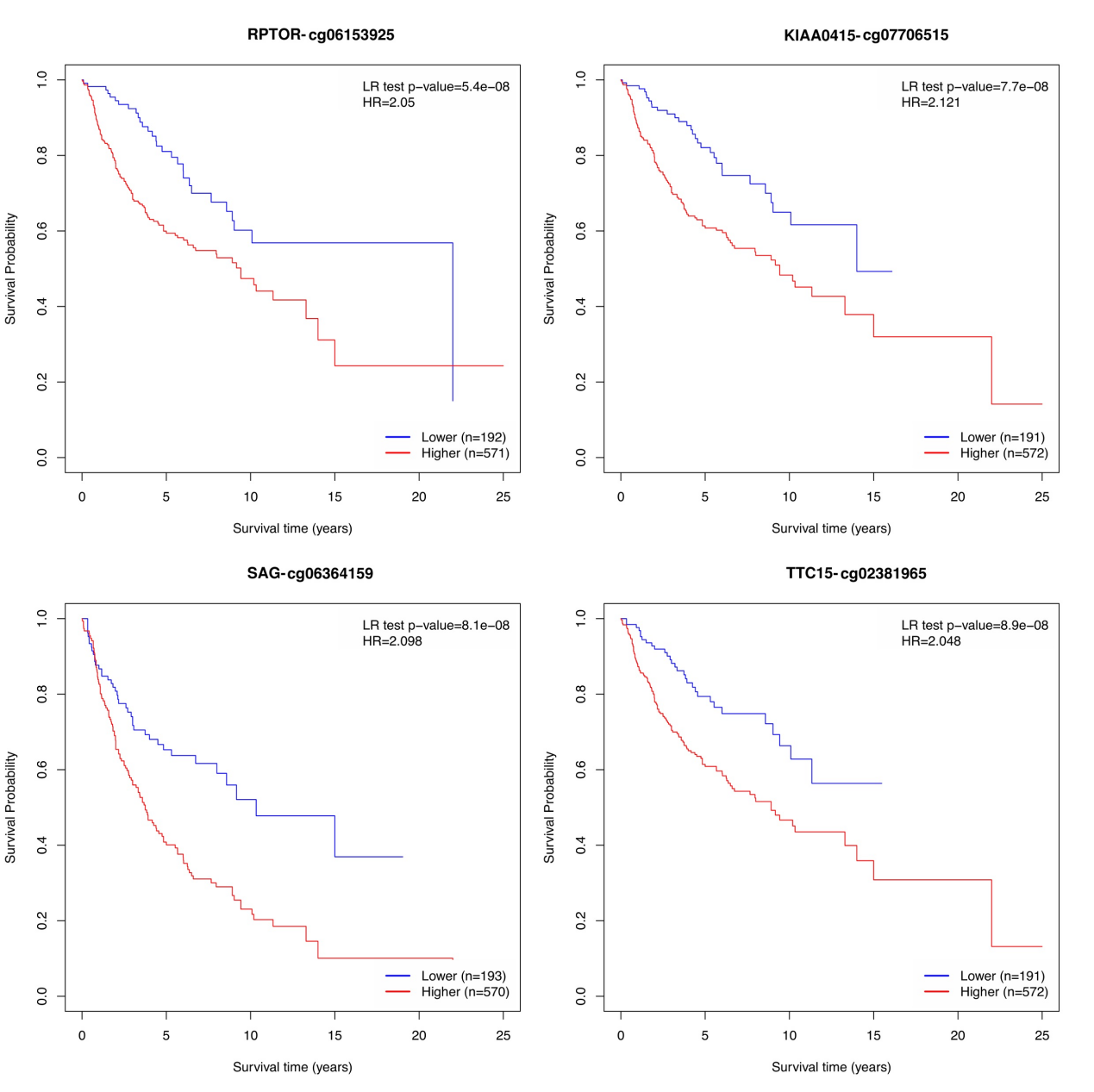


**Supplementary Figure S4.** Kaplan–Meier plots depicting the effect of other top four prediction biomarkers (log-rank test <0.05). Methylation groups are dichotomized by higher and lower methylation groups based on a cut-off point such as mean, median, or upper and lower quantiles. The X-axis denotes survival time in years and the Y-axis denotes the probability of patient survival.

**
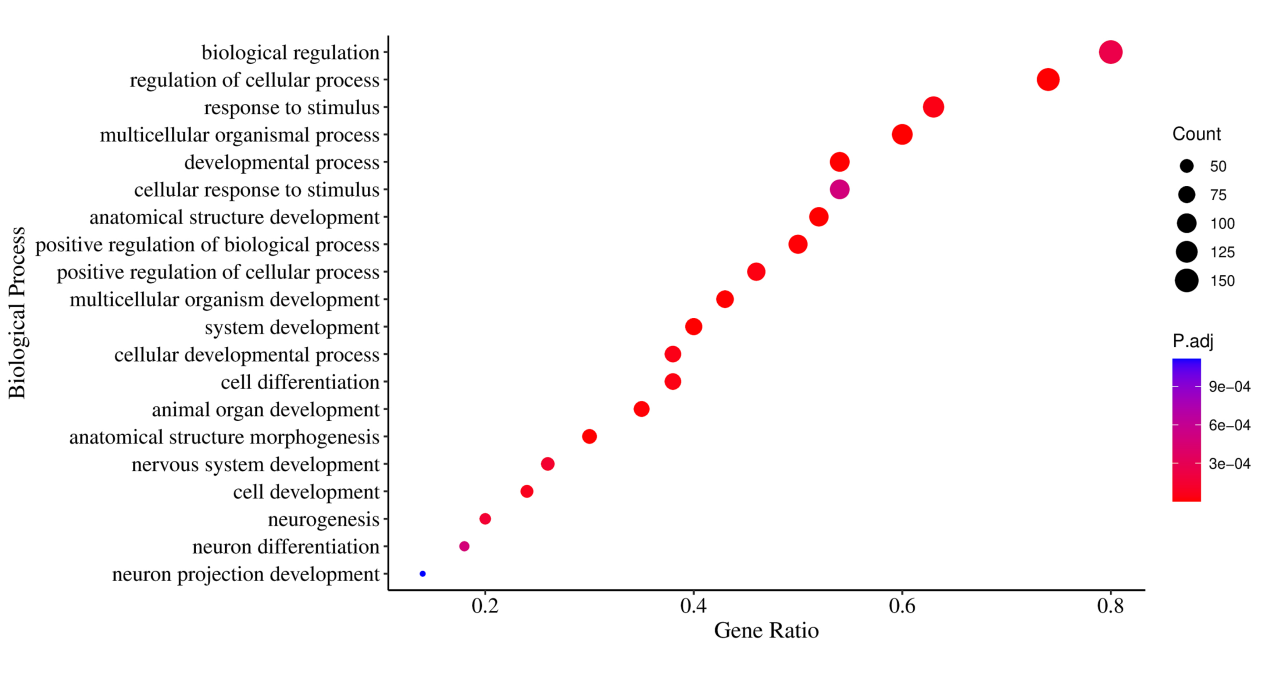
**

**Supplementary Figure S5.** Functional enrichment analysis results displayed as a dot plot**.** The top 20 enriched biological processes are shown on the Y-axis, and the X-axis indicates the ratio of enriched genes. The size of the dots reflects the number of enriched genes, and the color indicates the adjusted p-value. The largest dot represents the biological regulation process, with 87.7% enrichment, and 43 out of 49 genes in the nervous system development were also enriched in this process.


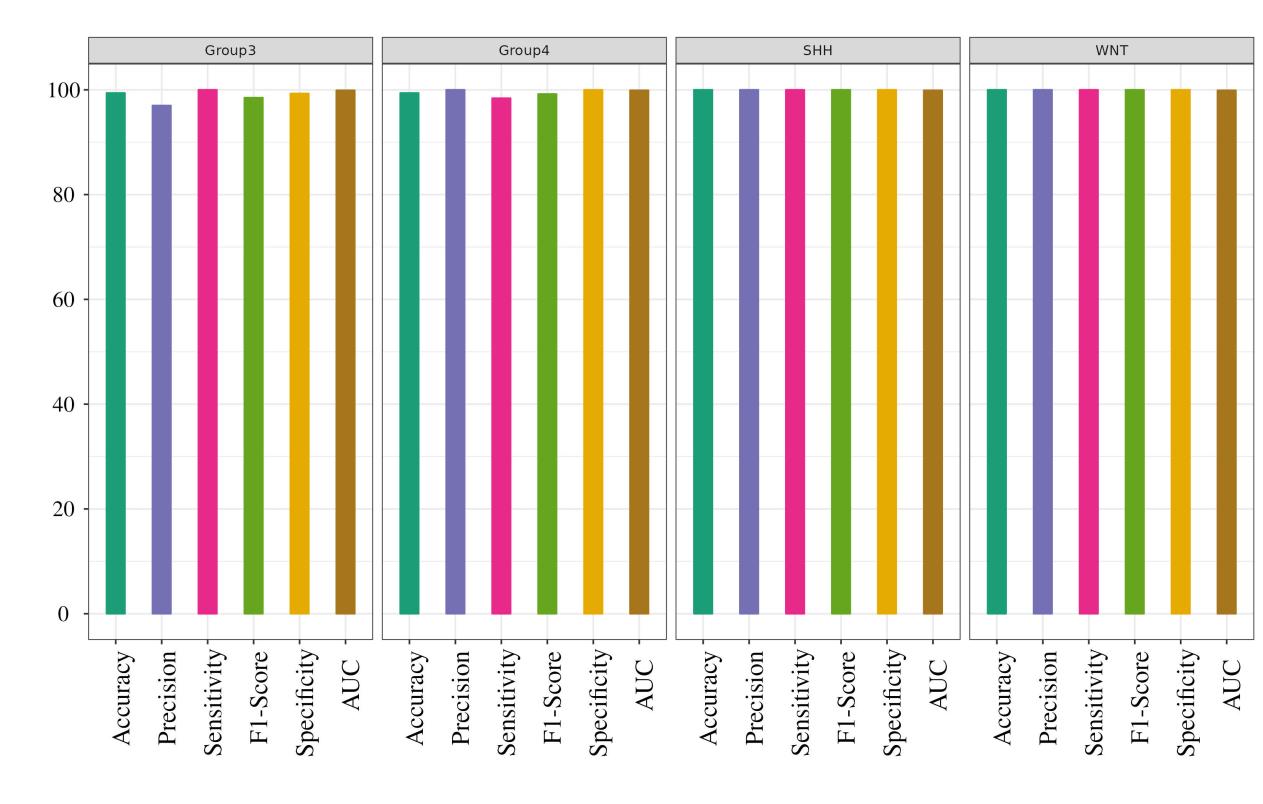


**Supplementary Figure S6**. Barplot displaying the performance metrics of the Artificial Neural Network (ANN) trained using enriched genes from the nervous system development process.

## **Supplementary Tables**

**Supplementary Table S1.** **Sample information and labels in the training dataset assigned through SNF and SC techniques**. The table presents the number of samples from the methylation data, along with the labels assigned using Similarity Network Fusion (SNF) and spectral clustering (SC) techniques, in comparison to the original labels.

| Samples | SC label | Original label |
| --- | --- | --- |
| GSM2261607 | Group3 | Group4 |
| GSM2261630 | Group3 | Group4 |
| GSM2261712 | SHH | WNT |
| GSM2261763 | Group4 | Group3 |
| GSM2261784 | Group3 | Group4 |
| GSM2261794 | Group4 | Group3 |
| GSM2261852 | Group3 | Group4 |
| GSM2261884 | Group3 | Group4 |
| GSM2261885 | WNT | Group3 |
| GSM2261935 | Group3 | Group4 |
| GSM2261951 | Group3 | Group4 |
| GSM2262053 | Group3 | Group4 |
| GSM2262175 | Group3 | Group4 |
| GSM2262192 | Group4 | Group3 |
| GSM2262253 | Group3 | Group4 |
| GSM2262312 | Group3 | Group4 |

**Supplementary Table S2.** Table displaying the confusion matrix obtained from all the prediction models using GSE90496 as the validation set.

|  | Model | Prediction | | | | |
| --- | --- | --- | --- | --- | --- | --- |
| Truth | RF | Subgroups | Group3 | Group4 | SHH | WNT |
|  |  | Group3 | 74 | 3 | 0 | 0 |
|  |  | Group4 | 3 | 135 | 0 | 0 |
|  |  | SHH | 0 | 0 | 136 | 0 |
|  |  | WNT | 0 | 0 | 0 | 39 |
|  | SVM | Group3 | 74 | 1 | 0 | 0 |
|  |  | Group4 | 4 | 134 | 0 | 0 |
|  |  | SHH | 0 | 0 | 136 | 0 |
|  |  | WNT | 0 | 0 | 0 | 39 |
|  | XGB | Group3 | 72 | 4 | 0 | 1 |
|  |  | Group4 | 3 | 135 | 0 | 0 |
|  |  | SHH | 0 | 0 | 136 | 0 |
|  |  | WNT | 0 | 0 | 1 | 38 |
|  | NB | Group3 | 75 | 2 | 0 | 0 |
|  |  | Group4 | 3 | 135 | 0 | 0 |
|  |  | SHH | 0 | 0 | 136 | 0 |
|  |  | WNT | 0 | 0 | 0 | 39 |
|  | LDA | Group3 | 69 | 7 | 0 | 1 |
|  |  | Group4 | 9 | 129 | 0 | 0 |
|  |  | SHH | 0 | 0 | 136 | 0 |
|  |  | WNT | 1 | 0 | 0 | 38 |
|  | KNN | Group3 | 74 | 3 | 0 | 0 |
|  |  | Group4 | 2 | 136 | 0 | 0 |
|  |  | SHH | 0 | 0 | 136 | 0 |
|  |  | WNT | 0 | 0 | 0 | 39 |
|  | ANN | Group3 | 75 | 2 | 0 | 0 |
|  |  | Group4 | 3 | 135 | 0 | 0 |
|  |  | SHH | 0 | 0 | 136 | 0 |
|  |  | WNT | 0 | 0 | 0 | 39 |

**Supplementary Table S3**. Table displaying the performance metrics of prediction models using GSE130051 dataset as the validation set.

| Subgroup | Accuracy | Precision | Sensitivity | F1-Score | Specificity | AUC | Model |
| --- | --- | --- | --- | --- | --- | --- | --- |
| Overall | 0.94 | 0.96 | 0.89 | 0.92 | 0.98 | 0.95 | RF |
| Group3 | 0.952 | 0.92 | 0.95 | 0.93 | 0.95 | 0.95 | SVM |
| Group4 | 0.95 | 0.97 | 0.95 | 0.96 | 0.96 | 0.95 |  |
| WNT | 0.99 | 0 | - | - | 0.99 | 0.95 |  |
| Group3 | 0.95 | 0.92 | 0.96 | 0.94 | 0.95 | 0.96 | XGB |
| WNT | 0.99 | 0 | - | - | 0.99 | 0.96 |  |
| Group4 | 0.96 | 0.98 | 0.95 | 0.96 | 0.96 | 0.96 |  |
| Overall | 0.96 | 0.99 | 0.92 | 0.95 | 0.99 | 0.97 | NB |
| Group3 | 0.91 | 0.88 | 0.86 | 0.87 | 0.93 | 0.90 | LDA |
| SHH | 0.99 | 0 | - | - | 0.99 | 0.90 |  |
| WNT | 0.99 | 0 | - | - | 0.99 | 0.90 |  |
| Group4 | 0.91 | 0.93 | 0.93 | 0.93 | 0.86 | 0.90 |  |
| Overall | 0.934 | 0.93 | 0.89 | 0.91 | 0.96 | 0.93 | KNN |
| Overall | 0.95 | 0.96 | 0.90 | 0.93 | 0.98 | 0.95 | ANN |

**Supplementary Table S4.** Table displaying the confusion matrix obtained from all the prediction models using GSE130051 as the validation set.

|  | Model | Prediction | | | | |
| --- | --- | --- | --- | --- | --- | --- |
| Truth | RF | Subgroups | Group3 | Group4 | WNT | SHH |
|  |  | Group3 | 471 | 17 | 1 |  |
|  |  | Group4 | 39 | 862 | 0 |  |
|  |  | WNT | 0 | 0 | 0 |  |
|  | SVM | Group3 | 472 | 17 | 1 |  |
|  |  | Group4 | 55 | 846 | 0 |  |
|  |  | WNT | 0 | 0 | 0 |  |
|  | XGB | Group3 | 469 | 19 | 1 |  |
|  |  | Group4 | 41 | 860 | 0 |  |
|  |  | WNT | 0 | 0 | 0 |  |
|  | NB | Group3 | 485 | 4 |  |  |
|  |  | Group4 | 42 | 859 |  |  |
|  | LDA | Group3 | 423 | 64 | 1 | 1 |
|  |  | Group4 | 56 | 845 | 0 | 0 |
|  |  | WNT | 0 | 0 | 0 | 0 |
|  |  | SHH | 0 | 0 | 0 | 0 |
|  | KNN | Group3 | 456 | 33 |  |  |
|  |  | Group4 | 54 | 847 |  |  |
|  | ANN | Group3 | 472 | 17 | 0 | 1 |
|  |  | Group4 | 54 | 847 | 0 | 0 |

**Supplementary Table S5**. Table displaying the performance metrics of prediction models using GSE54880 dataset as the validation set.

| Subgroup | Accuracy | Precision | Sensitivity | F1-Score | Specificity | AUC | Model |
| --- | --- | --- | --- | --- | --- | --- | --- |
| Group3 | 0.953 | 0.940 | 0.875 | 0.906 | 0.98 | 0.938 | RF |
| Group4 | 0.960 | 0.946 | 0.955 | 0.951 | 0.964 | 0.938 |  |
| SHH | 0.996 | 1 | 0.983 | 0.992 | 1 | 0.938 |  |
| WNT | 0.982 | 0.868 | 1 | 0.930 | 0.979 | 0.938 |  |
| Group3 | 0.957 | 0.955 | 0.875 | 0.913 | 0.985 | 0.937 | SVM |
| Group4 | 0.957 | 0.923 | 0.973 | 0.947 | 0.945 | 0.937 |  |
| SHH | 1 | 1 | 1 | 1 | 1 | 0.937 |  |
| WNT | 1 | 1 | 1 | 1 | 1 | 0.937 |  |
| Group3 | 0.964 | 0.956 | 0.903 | 0.929 | 0.985 | 0.972 | XGB |
| Group4 | 0.967 | 0.94 | 0.982 | 0.96 | 0.958 | 0.972 |  |
| SHH | 0.996 | 1 | 0.983 | 0.992 | 1 | 0.972 |  |
| WNT | 0.993 | 0.97 | 0.97 | 0.97 | 0.996 | 0.972 |  |
| Group3 | 0.971 | 0.932 | 0.958 | 0.945 | 0.975 | 0.957 | NB |
| Group4 | 0.978 | 0.973 | 0.973 | 0.973 | 0.982 | 0.957 |  |
| SHH | 1 | 1 | 1 | 1 | 1 | 0.957 |  |
| WNT | 0.993 | 1 | 0.939 | 0.969 | 1 | 0.957 |  |
| Group3 | 0.946 | 0.913 | 0.875 | 0.894 | 0.971 | 0.927 | LDA |
| Group4 | 0.953 | 0.93 | 0.955 | 0.942 | 0.952 | 0.927 |  |
| SHH | 0.996 | 0.984 | 1 | 0.992 | 0.995 | 0.927 |  |
| WNT | 0.996 | 1 | 0.97 | 0.985 | 1 | 0.927 |  |
| Group3 | 0.953 | 0.94 | 0.875 | 0.906 | 0.980 | 0.933 | KNN |
| Group4 | 0.953 | 0.922 | 0.964 | 0.943 | 0.945 | 0.933 |  |
| SHH | 1 | 1 | 1 | 1 | 1 | 0.933 |  |
| WNT | 1 | 1 | 1 | 1 | 1 | 0.933 |  |
| Group3 | 0.975 | 0.971 | 0.931 | 0.95 | 0.99 | 0.993 | ANN |
| Group4 | 0.975 | 0.956 | 0.982 | 0.969 | 0.97 | 0.993 |  |
| SHH | 1 | 1 | 1 | 1 | 1 | 0.993 |  |
| WNT | 1 | 1 | 1 | 1 | 1 | 0.993 |  |

**Supplementary Table S6.** Table displaying the confusion matrix obtained from all the prediction models using GSE54880 as the validation set.

|  | Model | Prediction | | | | |
| --- | --- | --- | --- | --- | --- | --- |
| Truth | RF | Subgroups | Group3 | Group4 | SHH | WNT |
|  |  | Group3 | 63 | 6 | 0 | 3 |
|  |  | Group4 | 4 | 106 | 0 | 1 |
|  |  | SHH | 0 | 0 | 59 | 1 |
|  |  | WNT | 0 | 0 | 0 | 33 |
|  | SVM | Group3 | 63 | 9 | 0 | 0 |
|  |  | Group4 | 3 | 108 | 0 | 0 |
|  |  | SHH | 0 | 0 | 60 | 0 |
|  |  | WNT | 0 | 0 | 0 | 33 |
|  | XGB | Group3 | 65 | 6 | 0 | 1 |
|  |  | Group4 | 2 | 109 | 0 | 0 |
|  |  | SHH | 0 | 1 | 59 | 0 |
|  |  | WNT | 1 | 0 | 0 | 32 |
|  | NB | Group3 | 69 | 3 | 0 | 3 |
|  |  | Group4 | 3 | 108 | 0 | 0 |
|  |  | SHH | 0 | 0 | 60 | 0 |
|  |  | WNT | 2 | 0 | 0 | 31 |
|  | LDA | Group3 | 63 | 8 | 1 | 0 |
|  |  | Group4 | 5 | 106 | 0 | 0 |
|  |  | SHH | 0 | 0 | 60 | 0 |
|  |  | WNT | 1 | 0 | 0 | 32 |
|  | KNN | Group3 | 63 | 9 | 0 | 0 |
|  |  | Group4 | 4 | 107 | 0 | 0 |
|  |  | SHH | 0 | 0 | 60 | 0 |
|  |  | WNT | 0 | 0 | 0 | 33 |
|  | ANN | Group3 | 67 | 5 | 0 | 0 |
|  |  | Group4 | 2 | 109 | 0 | 0 |
|  |  | SHH | 0 | 0 | 60 | 0 |
|  |  | WNT | 0 | 0 | 0 | 33 |

**Supplementary Table S7**. Table displaying the performance metrics of prediction models using GSE109379 dataset as the validation set.

| Subgroup | Accuracy | Precision | Sensitivity | F1-Score | Specificity | AUC | Model |
| --- | --- | --- | --- | --- | --- | --- | --- |
| Group3 | 0.984 | 0.917 | 1 | 0.957 | 0.981 | 0.966 | RF |
| Group4 | 0.977 | 1 | 0.93 | 0.964 | 1 | 0.966 |  |
| SHH | 0.984 | 0.964 | 1 | 0.982 | 0.973 | 0.966 |  |
| WNT | 0.992 | 1 | 0.889 | 0.941 | 1 | 0.966 |  |
| Group3 | 0.977 | 0.913 | 0.955 | 0.933 | 0.981 | 0.947 | SVM |
| Group4 | 0.969 | 0.976 | 0.93 | 0.952 | 0.988 | 0.947 |  |
| SHH | 0.984 | 0.964 | 1 | 0.982 | 0.973 | 0.947 |  |
| WNT | 0.992 | 1 | 0.889 | 0.941 | 1 | 0.947 |  |
| Group3 | 0.953 | 0.808 | 0.955 | 0.875 | 0.953 | 0.839 | XGB |
| Group4 | 0.969 | 0.976 | 0.93 | 0.952 | 0.988 | 0.839 |  |
| SHH | 0.992 | 0.982 | 1 | 0.991 | 0.986 | 0.839 |  |
| WNT | 0.961 | 0.833 | 0.556 | 0.676 | 0.922 | 0.839 |  |
| Group3 | 0.938 | 0.733 | 1 | 0.84 | 0.925 | 0.897 | NB |
| Group4 | 0.961 | 1 | 0.884 | 0.938 | 1 | 0.897 |  |
| SHH | 0.992 | 1 | 0.981 | 0.991 | 1 | 0.897 |  |
| WNT | 0.984 | 1 | 0.778 | 0.875 | 1 | 0.897 |  |
| Group3 | 0.922 | 0.8 | 0.727 | 0.76 | 0.962 | 0.828 | LDA |
| Group4 | 0.906 | 0.884 | 0.884 | 0.864 | 0.918 | 0.828 |  |
| SHH | 0.977 | 0.964 | 0.981 | 0.972 | 0.973 | 0.828 |  |
| WNT | 0.992 | 1 | 0.889 | 0.941 | 1 | 0.828 |  |
| Group3 | 0.953 | 0.864 | 0.864 | 0.864 | 0.972 | 0.899 | KNN |
| Group4 | 0.945 | 0.929 | 0.907 | 0.918 | 0.965 | 0.899 |  |
| SHH | 0.984 | 0.964 | 1 | 0.982 | 0.973 | 0.899 |  |
| WNT | 0.992 | 1 | 0.889 | 0.941 | 1 | 0.899 |  |
| Group3 | 0.977 | 0.913 | 0.955 | 0.933 | 0.981 | 0.981 | ANN |
| Group4 | 0.969 | 0.976 | 0.93 | 0.952 | 0.988 | 0.981 |  |
| SHH | 0.984 | 0.964 | 1 | 0.982 | 0.973 | 0.981 |  |
| WNT | 0.992 | 1 | 0.889 | 0.941 | 1 | 0.981 |  |

**Supplementary Table S8.** Table displaying the confusion matrix obtained from all the prediction models using GSE109379 as the validation set.

|  | Model | Prediction | | | | |
| --- | --- | --- | --- | --- | --- | --- |
| Truth | RF | Subgroups | Group3 | Group4 | SHH | WNT |
|  |  | Group3 | 22 | 0 | 0 | 2 |
|  |  | Group4 | 2 | 40 | 1 | 0 |
|  |  | SHH | 0 | 0 | 54 | 0 |
|  |  | WNT | 0 | 0 | 1 | 8 |
|  | SVM | Group3 | 21 | 1 | 0 | 2 |
|  |  | Group4 | 2 | 40 | 1 | 0 |
|  |  | SHH | 0 | 0 | 54 | 0 |
|  |  | WNT | 0 | 0 | 1 | 8 |
|  | XGB | Group3 | 21 | 1 | 0 | 0 |
|  |  | Group4 | 2 | 40 | 0 | 1 |
|  |  | SHH | 0 | 0 | 54 | 0 |
|  |  | WNT | 3 | 0 | 1 | 5 |
|  | NB | Group3 | 22 | 0 | 0 | 3 |
|  |  | Group4 | 5 | 38 | 0 | 0 |
|  |  | SHH | 1 | 0 | 53 | 0 |
|  |  | WNT | 2 | 0 | 0 | 7 |
|  | LDA | Group3 | 16 | 6 | 0 | 3 |
|  |  | Group4 | 4 | 38 | 1 | 0 |
|  |  | SHH | 0 | 1 | 53 | 0 |
|  |  | WNT | 0 | 0 | 1 | 8 |
|  | KNN | Group3 | 19 | 3 | 0 | 3 |
|  |  | Group4 | 3 | 39 | 1 | 0 |
|  |  | SHH | 0 | 0 | 53 | 0 |
|  |  | WNT | 0 | 0 | 1 | 8 |
|  | ANN | Group3 | 21 | 1 | 0 | 0 |
|  |  | Group4 | 2 | 40 | 1 | 0 |
|  |  | SHH | 0 | 0 | 54 | 0 |
|  |  | WNT | 0 | 0 | 1 | 8 |

**Supplementary Table S9**. Table displaying the confusion matrix obtained from all the prediction models using GSE75153 dataset as the validation set.

| Subgroup | Accuracy | Precision | Sensitivity | F1-Score | Specificity | AUC | Model |
| --- | --- | --- | --- | --- | --- | --- | --- |
| Group3 | 0.956 | 0.923 | 0.8 | 0.857 | 0.987 | 0.926 | RF |
| Group4 | 0.978 | 0.882 | 1 | 0.938 | 0.974 | 0.926 |  |
| SHH | 0.989 | 1 | 0.978 | 0.989 | 1 | 0.926 |  |
| WNT | 0.989 | 0.941 | 1 | 0.97 | 0.987 | 0.926 |  |
| Group3 | 0.978 | 0.933 | 0.933 | 0.933 | 0.987 | 0.97 | SVM |
| Group4 | 0.989 | 0.938 | 1 | 0.968 | 0.987 | 0.97 |  |
| SHH | 0.989 | 1 | 0.978 | 0.989 | 1 | 0.97 |  |
| WNT | 1 | 1 | 1 | 1 | 1 | 0.97 |  |
| Group3 | 0.978 | 0.933 | 0.933 | 0.933 | 0.987 | 0.989 | XGB |
| Group4 | 0.989 | 0.938 | 1 | 0.968 | 0.987 | 0.989 |  |
| SHH | 0.989 | 1 | 0.978 | 0.989 | 1 | 0.989 |  |
| WNT | 1 | 1 | 1 | 1 | 1 | 0.989 |  |
| Group3 | 0.967 | 0.875 | 0.933 | 0.903 | 0.974 | 0.955 | NB |
| Group4 | 0.989 | 0.938 | 1 | 0.968 | 0.987 | 0.955 |  |
| SHH | 0.989 | 1 | 0.978 | 0.989 | 1 | 0.955 |  |
| WNT | 0.989 | 1 | 0.938 | 0.968 | 1 | 0.955 |  |
| Group3 | 0.978 | 0.933 | 0.933 | 0.933 | 0.987 | 0.97 | LDA |
| Group4 | 0.989 | 0.938 | 1 | 0.968 | 0.987 | 0.97 |  |
| SHH | 0.989 | 1 | 0.978 | 0.989 | 1 | 0.97 |  |
| WNT | 1 | 1 | 1 | 1 | 1 | 0.97 |  |
| Group3 | 0.978 | 0.933 | 0.933 | 0.933 | 0.987 | 0.97 | KNN |
| Group4 | 0.989 | 0.938 | 1 | 0.968 | 0.987 | 0.97 |  |
| SHH | 0.989 | 1 | 0.978 | 0.989 | 1 | 0.97 |  |
| WNT | 1 | 1 | 1 | 1 | 1 | 0.97 |  |
| Group3 | 0.978 | 0.933 | 0.933 | 0.933 | 0.987 | 0.989 | ANN |
| Group4 | 0.989 | 0.938 | 1 | 0.968 | 0.987 | 0.989 |  |
| SHH | 0.989 | 1 | 0.978 | 0.989 | 1 | 0.989 |  |
| WNT | 1 | 1 | 1 | 1 | 1 | 0.989 |  |

**Supplementary Table S10.** Table displaying the confusion matrix obtained from all the prediction models using GSE75153 as the validation set.

|  | Model | Prediction | | | | |
| --- | --- | --- | --- | --- | --- | --- |
| Truth | RF | Subgroups | Group3 | Group4 | SHH | WNT |
|  |  | Group3 | 12 | 2 | 0 | 1 |
|  |  | Group4 | 0 | 15 | 0 | 0 |
|  |  | SHH | 1 | 0 | 44 | 0 |
|  |  | WNT | 0 | 0 | 0 | 16 |
|  | SVM | Group3 | 14 | 1 | 0 | 1 |
|  |  | Group4 | 0 | 15 | 0 | 0 |
|  |  | SHH | 1 | 0 | 44 | 0 |
|  |  | WNT | 0 | 0 | 0 | 16 |
|  | XGB | Group3 | 13 | 2 | 0 | 1 |
|  |  | Group4 | 0 | 15 | 0 | 0 |
|  |  | SHH | 1 | 0 | 44 | 0 |
|  |  | WNT | 0 | 0 | 0 | 16 |
|  | NB | Group3 | 14 | 1 | 0 | 0 |
|  |  | Group4 | 0 | 15 | 0 | 0 |
|  |  | SHH | 1 | 0 | 44 | 0 |
|  |  | WNT | 1 | 0 | 0 | 15 |
|  | LDA | Group3 | 14 | 1 | 0 | 0 |
|  |  | Group4 | 0 | 15 | 0 | 0 |
|  |  | SHH | 0 | 0 | 44 | 0 |
|  |  | WNT | 1 | 0 | 0 | 16 |
|  | KNN | Group3 | 14 | 1 | 0 | 0 |
|  |  | Group4 | 0 | 15 | 0 | 0 |
|  |  | SHH | 0 | 0 | 44 | 0 |
|  |  | WNT | 1 | 0 | 0 | 16 |
|  | ANN | Group3 | 14 | 1 | 0 | 0 |
|  |  | Group4 | 0 | 15 | 0 | 0 |
|  |  | SHH | 0 | 0 | 44 | 0 |
|  |  | WNT | 1 | 0 | 0 | 16 |

**Supplementary Table S11**. Enriched genes identified in the nervous system development process which was used for training the Artificial Neural Network (ANN) model.

| GO term | Enriched genes |
| --- | --- |
| Nervous system development | *ARHGEF7, ARTN, ASCL2, BOC, BTG2, CASZ1, CHRM3, CTNNA2, CUX1, CXCR4, DPYSL4, EP300, EPHA2, FES, FEZ2, IGFN1, KIDINS220, KIF5C, LEPR, LMTK2, LPAR3, LRP8, MEIS1, NFASC, NFIA, ORMDL3, PBX2, PLXNA2, PSD, RAP1GAP, RNF220, ROR1, RTN4RL1, S100A10, S100A8, SEMA4F, SHANK2, SKI, SLC8A1, SNTG2, SPTBN2, SSBP3, SYNGAP1, TLX2, TNFRSF1B, TRPV4, UGT8, WNT4, ZIC4* |

**Supplementary Table S12.** Comparison of MBMethPred with published studies in medulloblastoma Classification

| Method | Dataset | FS | Classifier | Samples | TFC_CpGs_ | SP | Acc |
| --- | --- | --- | --- | --- | --- | --- | --- |
| MBMethPred^*^ | Meth/  Gene | RF | RF, KNN, SVM, LDA, XGB, NB, ANN | 910 | 399 | WNT, SHH, G3, G4 | > 96% |
| Schwable et al. (2013) | Meth | - | SVM | 230 | 250 | WNT, SHH, G3, G4 | AUC < 80% |
| Schwable et al. (2017) | Meth | - | SVM, ANN, DT ,BN | 220 | 200 | WNT, SHH, G3, G4 | - |
| Gomez et al. (2018) | Meth | SSF, LDA | LDA | 913 /122 FFPE | 6 | WNT, SHH, G3, G4 | 92-100% |
| Korshunov et al. (2018) | RNA^1^/Meth | - | - | 239 | 22 | WNT, SHH, G3, G4 | - |
| Korshunov et al. (2019) | Meth | - | - | 78 | 10K | WNT | - |
| Capper et al. (2018) | Meth | RF | RF | 2,801^2^ | 10K | CNS tumors | AUC > 99% |
| Sharma et al. (2019) | Meth/  Gene | RF | RF | 1501 | 10K | G3 and G4 | AUC 0.9969 |
| Rathi et al. (2020)^*^ | Gene | Exp, VAR, BioF | GER | 173 | 1,399^$^ | WNT, SHH, G3, G4 | 85.71%- 100% |
| Attallah and Zaghlool, 2022 | Hist | TextA, DL | SVM, LDA, QDA, NB, KNN, RF | 154 images | - | Classic, Desmoplastic, Large cell, and Nodule | 100% |

^*^ Software package is available; ^$^Genes; ^1^ RNA-based NanoString; ^2^ CNS tumors; FS: Feature Selection; TFC: Total Features for Classification; SP: Subgroup Prediction; Acc; Accuracy; Meth: Methyation; Gene: Gene expression; Hist: Histopathological images; FFPE: Frozen and Formalin-Fixed Faraffin-Embedded; SSF: Stringent Selection Filters; RF: Random Forest; NB: Naive Bayes; KNN: K-Nearest Neighbor; SVM: Support Vector Machines; XGB: Extreme Gradient Boosting; LDA: Linear Discriminant Analysis; ANN: Artificial Neural Network; QDA: Quadratic Discriminant Analysis; BN: Bayesian Network; DT: Decision Tree; GER: Gene Expression Ratios; Exp: Expression; Var: Variance; BioF: Biotype Filters; TextA: Textural Analysis; DL: Deep learning; G3: Group 3; G4: Group 4

**Supplementary Data**

**Supplementary Data S1.** 399 CpG probes with their annotations and their beta values

**Supplementary Data S2.** Significant survival biomarkers with their statistical values and annotations
